# A review of the introduced herpetofauna of Mexico and Central America, with comments on the effects of invasive species and biosecurity methodology

**DOI:** 10.3897/zookeys.1022.51422

**Published:** 2021-03-08

**Authors:** Víctor Hugo González-Sánchez, Jerry D. Johnson, David González-Solís, Lydia Allison Fucsko, Larry David Wilson

**Affiliations:** 1 El Colegio de la Frontera Sur (ECOSUR), Chetumal, Quintana Roo, Mexico; 2 Department of Biological Sciences, The University of Texas at El Paso, El Paso, 79968-0500, TX, USA; 3 Department of Humanities and Social Sciences, Swinburne University of Technology, Melbourne, Victoria, Australia; 4 Centro Zamorano de Biodiversidad, Escuela Agrícola Panamericana Zamorano, Departamento de Francisco Morazán, Honduras;; 5 1350 Pelican Court, Homestead, 33035-1031, FL, USA

**Keywords:** Biological invasion, exotic species, herpetofauna, introduced species, Middle America, translocated species, Especies exóticas, especies introducidas, especies translocadas, herpetofauna, invasion biológica, mesoamérica

## Abstract

Among the principal causes producing detrimental effects on global biodiversity are introductions of alien species. Very few attempts to control introduced amphibians and reptiles in Middle America (Mexico and Central America) can be identified, so listings are provided for 24 exotic species, 16 translocated species, and 11 species that were removed from the introduced species listing because of lack of substantiating evidence that they are from established populations. Biosecurity methods are also identified that can be applied for preventing, controlling, and managing introduced and especially invasive species.

## Abstract (es)

Entre las principales causas que producen efectos perjudiciales sobre la biodiversidad mundial se encuentran la introducción de especies exóticas. Se pueden identificar muy pocos intentos de controlar anfibios y reptiles introducidos en América Central (México y América Central), por lo que proporcionamos listas de 24 especies exóticas, 16 especies translocadas y 11 especies que eliminamos de la lista de especies introducidas debido a la falta de evidencia que corrobore que provienen de poblaciones establecidas. También identificamos métodos de bioseguridad que se pueden aplicar para prevenir, controlar y gestionar especies introducidas y especialmente invasoras.

## Introduction

Among the most important drivers for biotic extinctions are introduced invasive species (Mack et al. 2000; Simberloff 2005). This phenomenon is not new, since human-mediated biological invasions or translocations of non-native species (e.g., goats, pigs, cats, dogs, and rats, among various mammals) by humans have been occurring for at least 20,000 years (Hofman and Rick 2018). Geographic scope, frequency, and number of species involved, however, have increased as a direct consequence of growths in transportation and commercial activities, so few habitats on Earth remain devoid of species introduced by humans (Mack et al. 2000). Among others, the main pathways for introducing amphibians and reptiles are accidental transport in cargo shipments on land and over water; intentional and accidental release via pet trade; as biocontrol agents; those associated with human food consumption, as well as for aesthetic purposes (Kraus 2003). Among many other effects, invasive non-native species can have negative impacts at all biological levels, including genetic pollution and hybridization (Cedeño-Vázquez et al. 2008), competition and depredation (Sakai et al. 2001), introducing parasites (Williams et al. 2013), epizootics (Garner et al. 2006), zoonoses (Hulme 2014), habitat modification, by altering nutrient and energy cycles and biomass structure (Beard et al. 2002; Crooks 2002), shifting water cycles (Gallardo et al. 2015), and triggering trophic cascades (Zavaleta et al. 2001). The impact of invasive species, such as the anurans *Lithobates catesbeianus* and *Rhinella horribilis*, as two well-known examples, on human economics and human well-being is considered collateral damage, because of the loss and alterations to goods (agricultural crops, animal husbandry, forest products, fisheries) and services (clean water, climate stabilization, pollination, human culture, recreation), as identified by Pejchar and Mooney (2009) and Walsh et al. (2016).

A common misbelief is that the ecology of invasive species and the ecosystem alterations they produce are extensively documented (Cadotte 2006). This is only partially true, since most research on ecology of alien species involves significant taxonomic and geographical biases (Pyšek et al. 2008; MacIsaac et al. 2010). This situation is relevant since historically introduced amphibians and reptiles have received less attention in Mesoamerica than have other groups, such as mammals, vascular plants, and insects (Reed and Kraus 2010). This deficiency has led to an omission of the status of introduced and invasive species in several herpetofaunal inventories. Therefore, listings of exotic and invasive herpetofaunal species in Middle America (Mexico and Central America) have been comprised of only a few species, including members of the genus *Boa*, Morelet’s Crocodile (*Crocodylus moreletii*), the Pond Slider (*Trachemys scripta*), and the American Bullfrog (*Lithobates catesbeianus*). Gekkonid lizards are found frequently in listings of introduced species, but their ecology and potential impacts have received little attention. Presently, 78 amphibian and 198 recognized reptile species have become established outside their native ranges around the world (Capinha et al. 2017). The goal of this paper is to review and assess the current knowledge and status of members of the introduced herpetofauna in Middle America, their history of colonization, their impacts on ecosystems, and their current geographic distributions.

## Materials and methods

We compiled a list of the introduced reptiles and amphibians in Middle America by examining relevant literature for the region, complemented with records obtained from GBIF (GBIF.org 2018), iNaturalist (inaturalist.org 2018), Amphibian Species of the World (Frost 2020), Reptile Database (Uetz et al. 2020), International Union for Conservation of Nature (2020), and VertNet (vertnet.org, 2018) platforms. In addition to these sources, for Mexico we considered only records with acceptable confirmation, properly georeferenced, and found in the literature and databases of the Comisión Nacional para el Conocimiento y Uso de la Biodiversidad (CONABIO 2018) and Secretaría de Medio Ambiente y Recursos Naturales (SEMARNAT 2017). Scientific names are based on Wilson et al. (2013a, b) and Johnson et al. (2015b), along with the most recent lists at Uetz et al. (2020) and Frost (2020), with full understanding that nomenclatural changes will occur regularly during future taxonomic revisions. Common names, when appropriate, follow Liner and Casas-Andreu (2008) for Mexico and adjacent Central America when species are shared between the two regions. Common names of species occurring only outside of Mexico and adjacent areas are those found in the literature and websites listed above. For convenience, we use the term “reptiles” to name taxonomic groups traditionally considered orders of the class Reptilia as listed by Uetz et al. (2020) (i.e.., Testudines – turtles; Crocodylia – crocodiles; Squamata – snakes and lizards), so we use the term herpetofauna when generally referring to the amphibians, turtles, crocodiles, snakes, and lizards occurring within Middle America. For more on our taxonomic positions, see the section below and especially Johnson et al. (2015b).

Our study area comprises Mexico and all Central American countries (Belize, Guatemala, Honduras, El Salvador, Nicaragua, Costa Rica, and Panama), ordered by latitude. Our use of the term “Middle America” refers to the Central American countries plus Mexico. We do not use the term “Mesoamerica,” since this label is generally considered more relevant in an anthropological and historical context (Romero-Contreras and Ávila-Ramos 1999), even though it is a commonly used synonym for Middle America.

In this paper, we consider introduced species to be populations whose presence in an area is attributed to human activities that enabled them to overcome biogeographical barriers that they otherwise could not cross and become established. We prefer introduced as the universal term over some others, such as exotic, non-native, alien, or non-indigenous, since the word “introduced” is more easily associated with human intervention (Young and Larson 2011). We will use the terms exotic and translocated most often, however, to describe the two major groupings of introduced species, and use other synonymous terms occasionally to lessen redundancy. This idea is clearly opposite to the concept of “native species,” to designate those that have evolved in a given area without human involvement, or that have arrived there by natural means without intentional or unintentional intervention of humans, from areas in which they are also native (Pyšek and Richardson 2010).

Introduced species are not all-encompassing on temporal and geographical bases, because not all introduced organisms manage to become “established;” i.e., surviving long enough to produce descendant lineages (Davis 2009). We also understand that some species occasionally can be “naturalized,” which are those introduced species sustaining self-replacing populations for several life cycles without, or despite, direct intervention by humans (Richardson et al. 2011). In this sense, “population persistence” is the ongoing accumulation of establishment successes by individuals arising within an area (Davis 2009). Non-native species do not have to be introduced necessarily within the totality of a region, but transported to areas within the same area outside their native range; these will be referred to as “translocated species” (Shine et al. 2000). Countries are good examples of useful geographic units for qualifying which type of introduction a population exemplifies (exotic versus translocated), although sometimes adjustments need to be made to meet criteria, such as between islands and the mainland belonging to the same country, or a slightly disconnected mainland from a large peninsula belonging to the same country (e.g., Baja California peninsula and mainland Mexico). A closely related concept is “invasive species,” here defined as those well-established introduced species having deleterious effects on native ecosystems as the result of increasing their population numbers. In addition, invasive species can have deleterious effects on native populations, even if their populations are not increasing, e.g., through the introduction of new diseases, which can then kill native species.

The “impact” of an introduced invasive species, either exotic or translocated, refers to how an introduced species distresses the physical, chemical, or biological environment, the effect of which might be evident at the genetic, individual, population, community, ecosystem, landscape, regional, or global levels (Parker et al. 1999; Mack et al. 2000; Richardson et al. 2011). Of course, we do not overlook the fact that impacts and the reasons for them might be controversial (Parker et al. 1999; Young and Larson 2011), and that many invasive species can have negative influences on cultural, economic, and social issues relating to alleged human welfare (McNeely 2001; Perrings et al. 2005; Pejchar and Mooney 2009). Those topics remain unexplored in the field of invasive species in the herpetological literature, so some of the invasive species covered herein will focus our discussions on known impacts affecting ecological components. Finally, we adopt the term “biosecurity,” as defined by Pyšek and Richardson (2010), as the management of risks posed by organisms to the economy, environment, and human health through exclusion (prevention of initial introduction), mitigation, adaptation, control, and eradication.

Our taxonomic positions follow those discussed in Johnson et al. (2010), Porras et al. (2013), and expanded upon by Johnson et al. (2015b), which are predicated on modern phylogenetic principles. We regard species as separate evolutionary lineages and consider them the lowest evolutionary lineage segment placed on a phylogeny in a formal phylogenetically based taxonomy. We also consider the subspecies category to be a taxonomic anachronism that should not be used in a formal classification hierarchy because subspecies merely represent geographic variations in populations of the same species that are connected by gene flow (intergradation) and not separate evolutionary lineages. We do, however, concede that officially classifying subspecies had much influence in the past, so we reference them in a historical perspective in a few taxa discussed below. In those cases, the taxonomy reverts to the binomial.

## Results and discussion

### Status of the exotic and translocated herpetofauna of Middle America

Currently, 40 species of herpetofaunal species are considered introduced to a region of Middle America, or indigenous to a region, but translocated to non-native areas. Of these, 24 are exotics (Tables 1, 2), whereas 16 are translocated (Tables 1, 3). Most are reptiles (30 [18 exotics and 12 translocations], and ten are anuran amphibians [six exotics and four translocations]). Three species are listed among the 100 worst invasive alien species of the world (Lowe et al. 2000): the American Bullfrog (*Lithobates catesbeianus*), the Puerto Rican Coqui (*Eleutherodactylus coqui*), and the Pond Turtle (*Trachemys scripta*). Twenty-six species are listed in the IUCN Red List of Threatened Species (IUCN 2014), with most of those, as expected, in the Least Concern category. Two, however, are under risk categories: The Spiny Chuckwalla (*Sauromalus hispidus*) as Near Threatened, and the Mexican Giant Musk Turtle (*Staurotypus triporcatus*) as Lower Risk/Near Threatened. Mexico has the largest number of introduced species (24; 13 exotics and 11 translocations), whereas El Salvador only has two species, both exotic. In addition to Mexico, Honduras also has translocated species, such as *Ctenosaura similis* on Isla Roatán (Table 1).

**Table 1.** List of introduced and translocated herpetofauna in the countries from Middle America. Introduced herpetofauna in Middle America (Right total = number of countries in which the species has “exotic” or “translocated” status; Bottom total = number of exotic and translocated species in that country). Parentheses enclose the number of exotic and translocated species for that taxon.

Taxa	Exotic (E) or translocated (T)	Mexico	Guatemala	Belize	Honduras	El Salvador	Nicaragua	Costa Rica	Panamá	Total	Main references
**Amphibians (10)**											
**Anurans (10)**											
**Eleutherodactylidae (4)**											
*Eleutherodactylus antillensis*	E								+	1	de Sousa et al. (1989), Barker and Rodríguez-Robles (2017)
*Eleutherodactylus coqui*	E							+			Barrantes-Madrigal (2017), Barrantes-Madrigal et al. (2019)
*Eleutherodactylus johnstonei*	E								+	1	Ibañez and Rand (1990), McCranie and Valdez-Orellana (2014)
*Eleutherodactylus planirostris*	E	+			+		+	+	+	5	Crawford et al. (2011), McCranie and Gutsche (2014), Barquero and Araya (2016), Cedeño-Vázquez et al. (2014), Alvarez-Romero et al. (2008)
**Hylidae (2)**											
*Osteopilus septentrionalis*	E							+		1	Savage (2002)
*Smilisca baudinii*	T	+								1	Recuero et al. (2004)
**Pipidae (1)**											
*Xenopus laevis*	E	+								1	Murphy (1983) Álvarez-Romero (2008), Peralta-García et al. (2014)
**Ranidae (3)**											
*Lithobates berlandieri*	T	+								1	Rorabaugh and Servoss (2006)
*Lithobates catesbeianus*	T	+								1	Casas-Andreu et al. (2001), Lemos-Espinal and Smith (2016), Grismer (2002)
*Lithobates forreri*	T	+								1	Grismer (2002)
**Reptiles (31)**											
**Crocodylia (1)**											
**Crocodylidae (1)**											
*Crocodylus moreletii*	T	+								1	Alvarez-Romero (2008)
**Squamata (23)**											
**Dactyloidae (4)**											
*Anolis allisoni*	E	+		+	+					3	Charruau et al. (2015), Schmidt (1941), McCranie and Köhler (2015), Glor et al. (2005)
*Anolis carolinensis*	E	+								1	Terán-Juárez et al. (2015)
*Ctenonotus cristatellus*	E							+		1	Savage (2002), Mayer (2010)
*Norops sagrei*	E	+	+	+	+			+	+	6	Lee (1996), Sexton and Brown (1977), Stuart (1955), Savage and Bolaños-Vives (2005), Batista et al. (2019)
**Gekkonidae (9)**											
*Gehyra mutilata*	E	+								1	Álvarez-Romero (2008) Cruz-Sáenz et al. (2017), Reynoso (1990a), Lemos-Espinal and Dixon (2013)
*Gekko gecko*	E			+						1	Meerman and Garel (2002)
*Hemidactylus frenatus*	E	+	+	+	+	+	+	+	+	8	Weterings and Vetter (2017)
*Hemidactylus garnotii*	E		+					+		2	Savage (2002), Morales et al. (2017)
*Hemidactylus haitianus*	E				+				+	2	McCranie (2015), Auth (1994)
*Hemidactylus mabouia*	E	+			+			+	+	4	Álvarez-Romero (2008), Gutsche and McCranie (2009), Abarca and Monge (2007), Auth (1994)
*Hemidactylus turcicus*	E	+							+	2	Martínez-Hernández et al., (2017), McCoy (1970), Lee (1996)
*Lepidodactylus lugubris*	E	+					+	+	+	4	Smith and Grant (1961), Savage (2002), Hoogmoed and Avila-Peres (2015)
*Tarentola mauritanica*	E	+									Ortíz-Mena et al. (2019)
**Iguanidae (5)**											
*Ctenosaura conspicuosa*	T	+								1	Grismer (2002)
*Ctenosaura pectinata*	T	+								1	Aguirre-Léon and Matías-Ferrer (2017)
*Ctenosaura similis*	T				+					1	McCranie and Valdés-Orellana (2014)
*Sauromalus hispidus*	T	+								1	Grismer (2002), Petren and Case (1997)
*Sauromalus varius*	T	+								1	Hollingsworth et al. (1997)
**Leiocephalidae (1)**											
*Leiocephalus varius*	E				+					1	Schwartz and Thomas (1975), McCranie (2018)
**Phrynosomatidae (1)**											
*Uta stansburiana*	T	+								1	Upton and Murphy (1997)
**Sphaerodactylidae (1)**											
*Sphaerodactylus argus*	E	+					+		+	3	Harris and Kluge (1984), Lee (1996), Sunyer et al. (2013), Thomas (1975)
**Boidae (1)**											
*Boa imperator*	T	+								1	López-González (1991)
**Typhlopidae (1)**											
*Indotyphlops braminus*	E	+	+	+	+	+	+			6	Wallach et al. (2014), Leets-Rodríguez et al. (2019)
**Testudines (7)**											
**Chelydridae (1)**											
*Chelydra serpentina*	E	+								1	Dixon (2013), Powell et al. (2016), Terán-Juárez et al. (2016)
**Emydidae (1)**											
*Trachemys scripta*	T	+								1	Lavín et al. (2014)
**Kinosternidae (1)**											
***Kinosternon integrum***	T	+								1	Iverson et al. (1998)
**Staurotypidae (1)**											
*Staurotypus triporcatus*	T	+								1	Terán-Juárez et al. (2015)
**Testudinidae (1)**											
*Chelonoidis carbonarius*	E						+				Salazar-Saveedra et al. (2015), Villa et al. (1988)
**Trionychidae (1)**											
*Apalone spinifera*	T	+								1	Grismer (2002), McGaugh and Janzen (2008)
**Totals**	24E/16T	29	4	5	9	2	6	9	10	–	

Herpetofaunal introductions in Middle America can be traced back to colonial times and were associated with commercial routes between the Philippines and New Spain (mainly Acapulco, Mexico), or through the slave trade from Western Africa to the Caribbean and Antillean islands, and from there into Central America. More recent events involved the opening of the Panama Canal in 1914, the expansion of the irrigation infrastructure after the 1950’s in northern Mexico, and throughout Middle America due to the highly popular pet trade and agricultural practices. On the other hand, translocations have more obscure origins, and certainly some of those could have occurred in pre-Columbian times, like translocations of iguanid lizards onto several islands in the Sea of Cortes (also called the Gulf of California, or Mar de Cortés in Spanish) by the Seri society. Whereas it is often accepted in invasive species biology that 1492 is the cutoff date for delineation between native and non-native species, we are concerned at this point with translocated species, not non-native species. In addition, it is our opinion that the year 1492, as the time when Cristopher Columbus “discovered” the New World, is of disputable significance from a biological point of view. The matter of most significant concern, we think, is to what extent humans, whether from Spain or elsewhere, have had a hand in the movement of creatures around the world.

**Figure 1.**
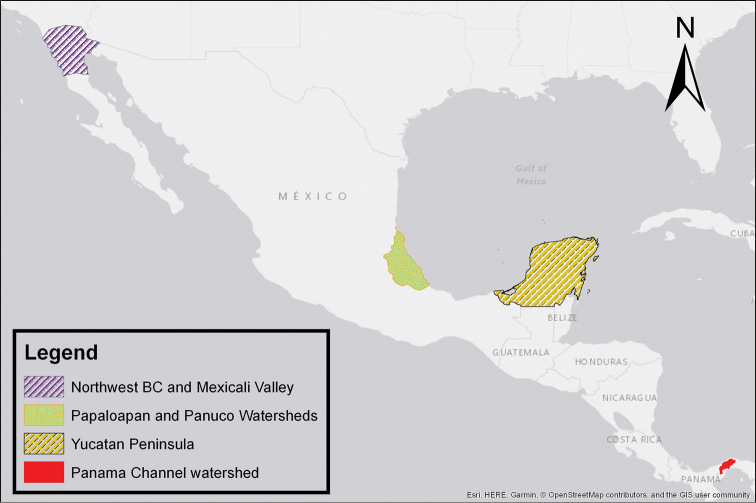
The four main Middle American sites considered as “hotspots” for herpetofaunal invasions: Northwestern Baja California and nearby Río Colorado delta in the Mexicali Valley, the Panama Canal, the Mexican Yucatan Peninsula, and The Papaloapan and Panuco basins in the Mexican state of Veracruz. The airports and seaports are not depicted due their ubiquity.

We recognize six major Middle American sites as “hotspots” for herpetofaunal invasions (four of which are depicted on Fig. 1): 1) Northwestern Baja California and nearby Río Colorado delta in the Mexicali Valley, where hydrological systems and agricultural channels most likely served as pathways for the invasion of several introduced species already established in California, Arizona, and New Mexico; 2) The Panama Canal, where concentrated traffic of cargo shipping has been an important source of invaders; 3) The Mexican Yucatan Peninsula, a tropical region with flat topography and low elevation, with its highest point around 300 m, containing extensive communication and tourism infrastructures; 4) The State of Veracruz, historically having the largest and most important Mexican sea port, as well as being a main logistic center for Mexico’s economy; 5) Major airports and seaports of each Middle American country; and 6) Insular systems within the Sea of Cortes and Pacific Ocean in northwestern Mexico, where distributional patterns of several species can be explained only by natural or translocated dispersal overwater and island hopping; some translocations could have occurred there during pre-Columbian or even prehistoric times.

Finally, we recognize the following four major causes of introductions: 1) accidentals, mainly small species transported inadvertently by cargo vehicles, most frequently gekkonid lizards and anurans; 2) intentional releases, principally associated with pet trade and as food resources; most significantly chelonians and iguanid lizards, respectively; 3) escapees from the farming industry; mainly crocodiles and anurans, such as Morelet’s Crocodiles and American Bullfrogs; and 4) expanding invasion fronts when introduced naturalized species with high reproduction potential are well adapted to altered habitats. This fourth mechanism is especially relevant in anurans, such as those within the genus *Eleutherodactylus* and other species of original dispersers along invasion fronts.

### The exotic herpetofauna of Middle America

#### Amphibia – Anura – Frogs

**Family Eleutherodactylidae**

***Eleutherodactylus antillensis* (Reinhardt & Lütken, 1863)**

The Antilles Robber Frog is native to Puerto Rico, the Virgin Islands, and several associated islands and cays in that region. Numerous individuals apparently were introduced in the late 1950’s or early 1960’s into Panama City, probably through ornamental plants or intentionally released by a family after returning from a trip to Puerto Rico (Barker and Rodríguez-Robles 2017). Since then, this frog has spread throughout suburban and rural gardens, abandoned parcels, and pastures in the Panama City metropolitan area (Barker and Rodríguez-Robles 2017) (Table 2, Map 1).

**Table 2.** Distribution of introduced amphibians and reptiles in Mexico and Central American countries.

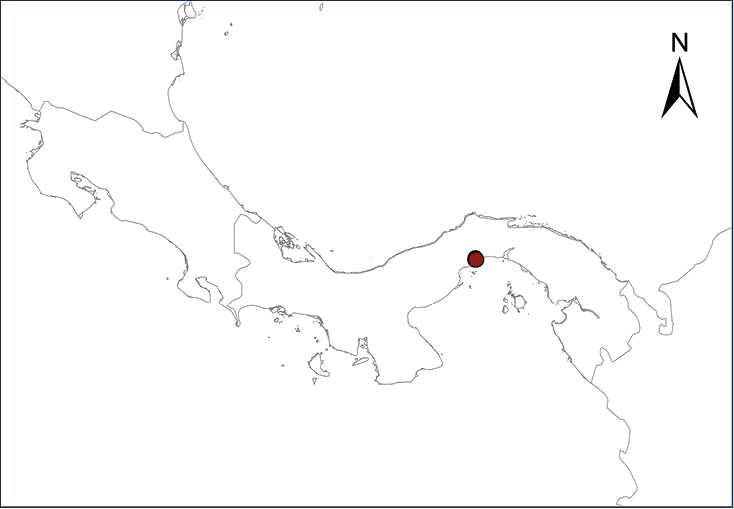	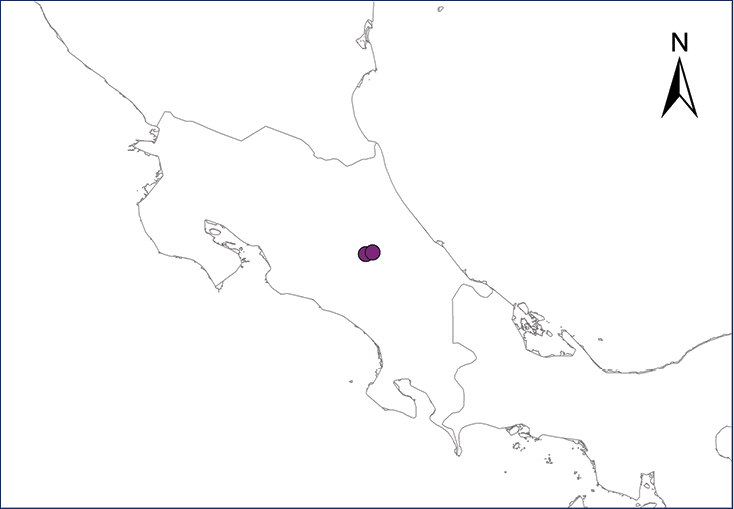
**Map 1.***Eleutherodactylus antillensis*.	**Map 2.***Eleutherodactylus coqui*.
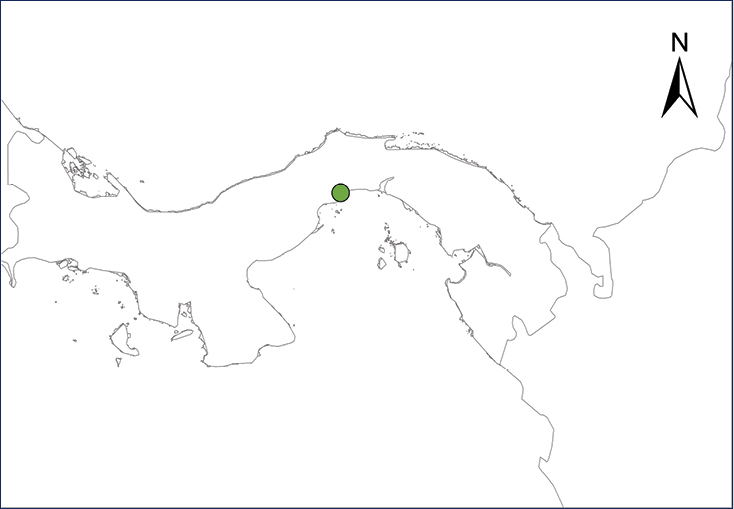	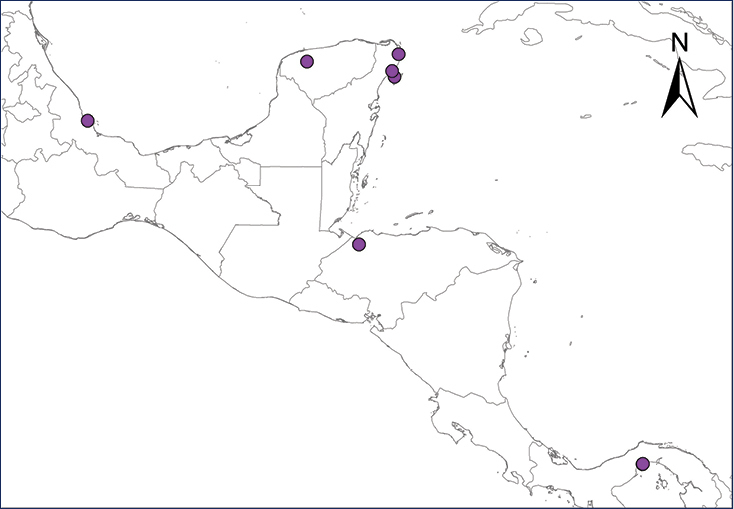
**Map 3.***Eleutherodactylus johnstonei*.	**Map 4.***Eleutherodactylus planirostris*.
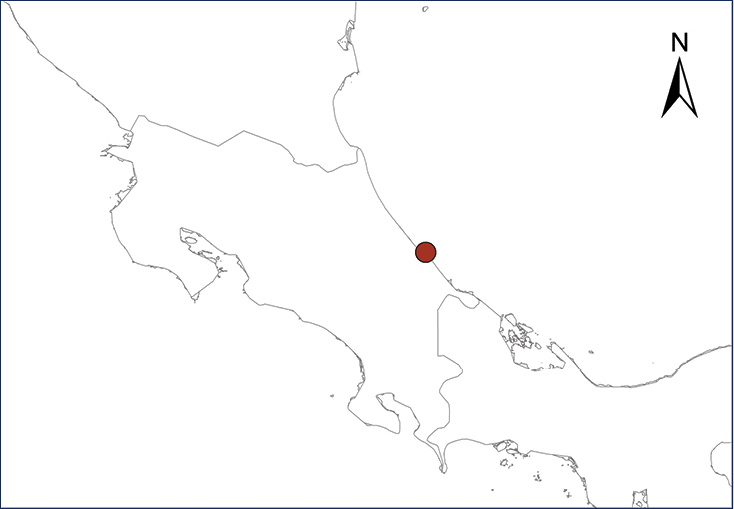	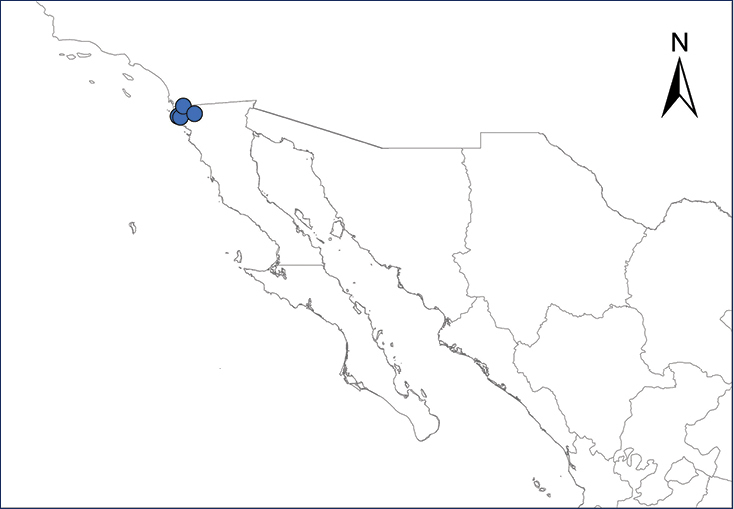
**Map 5.***Osteopilus septentrionalis*.	**Map 6.***Xenopus laevis*.
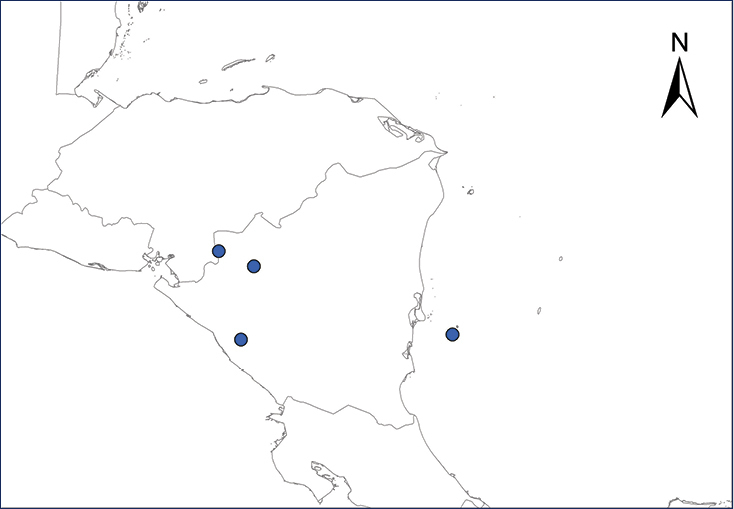	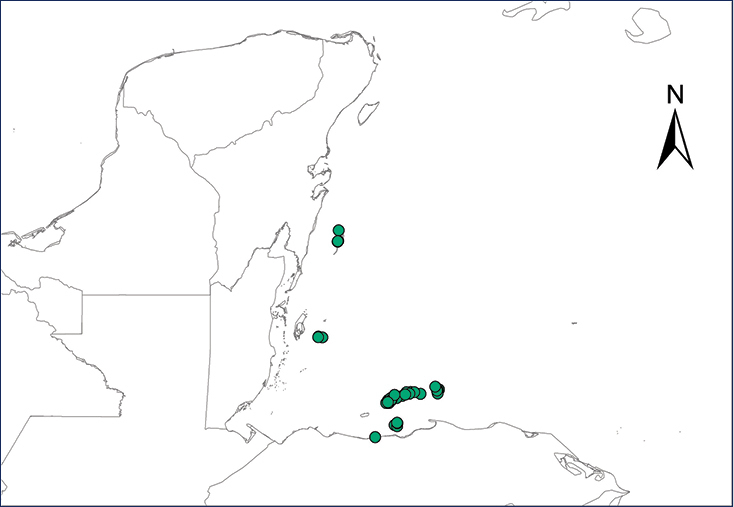
**Map 7.***Chelonoidis carbonarius*.	**Map 8.***Anolis allisoni*.
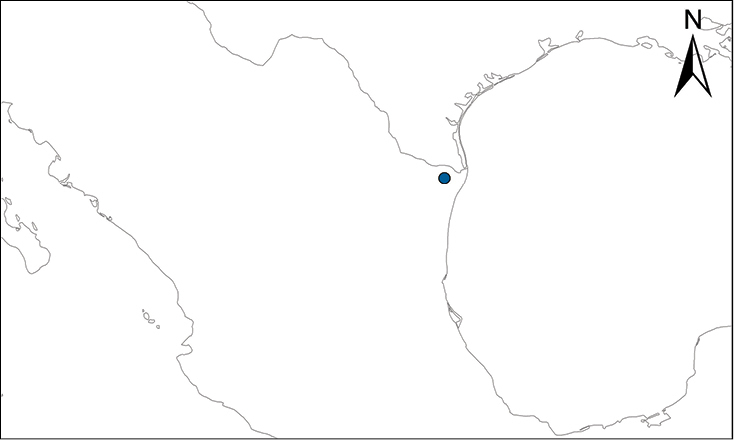	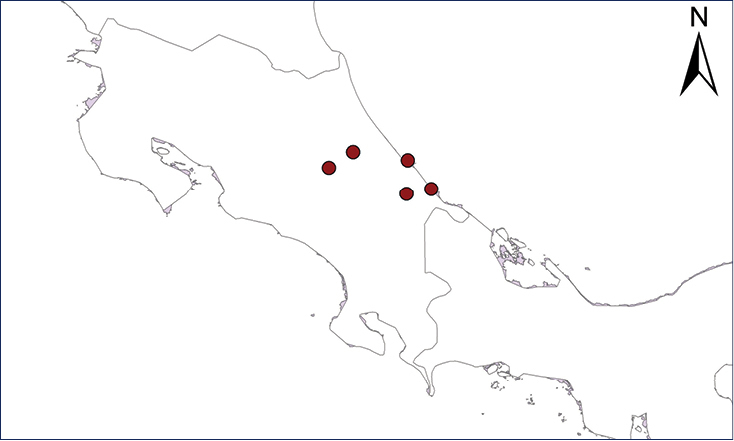
**Map 9.***Anolis carolinensis*.	**Map 10.***Ctenonotus cristatellus*.
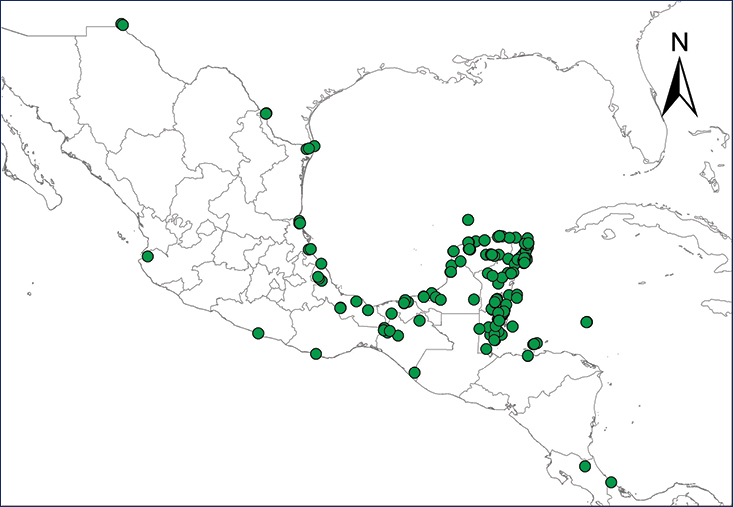	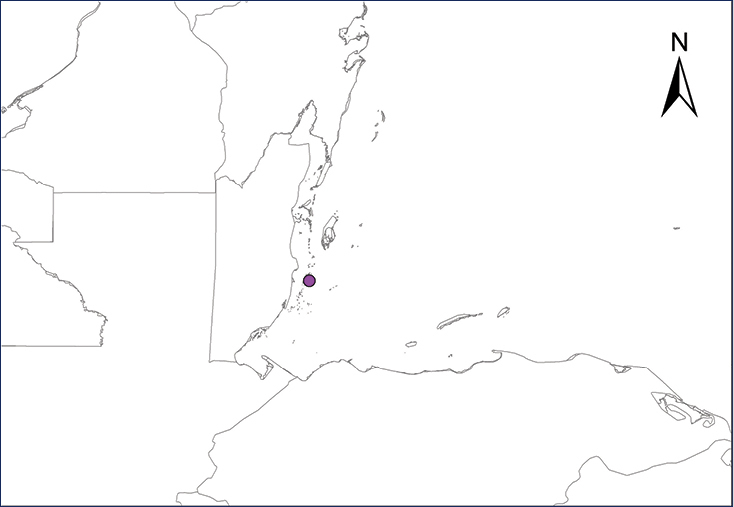
**Map 11.***Norops sagrei*.	**Map 12.***Gekko gecko*.
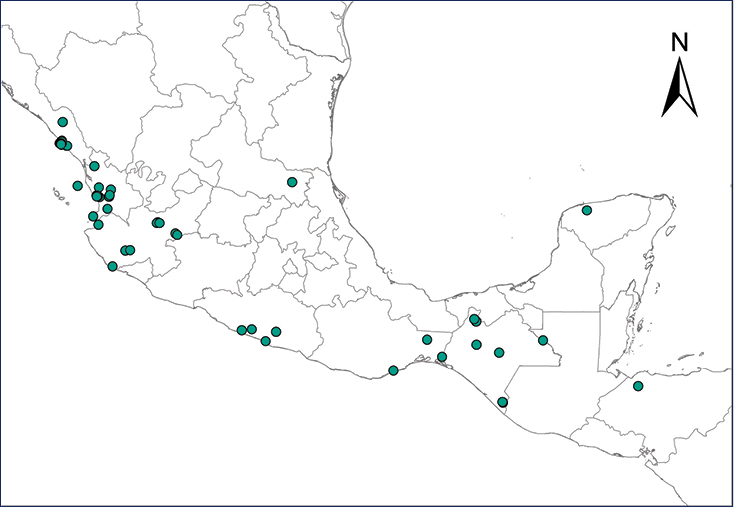	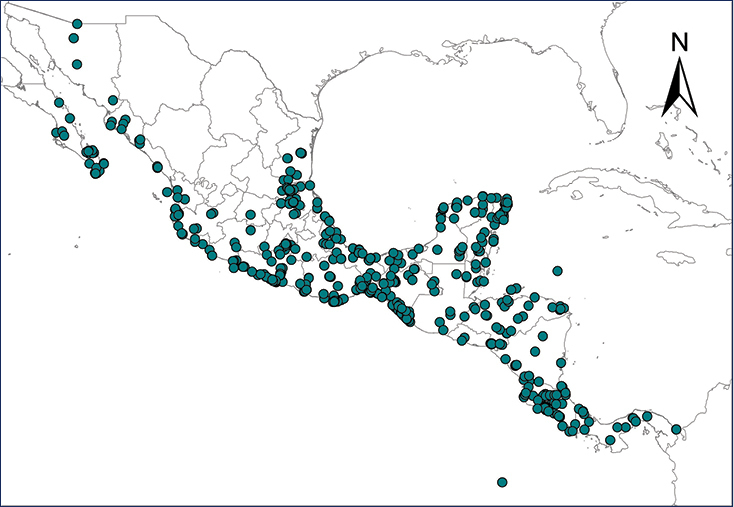
**Map 13.***Gehyra mutilata*.	**Map 14.***Hemidactylus frenatus*.
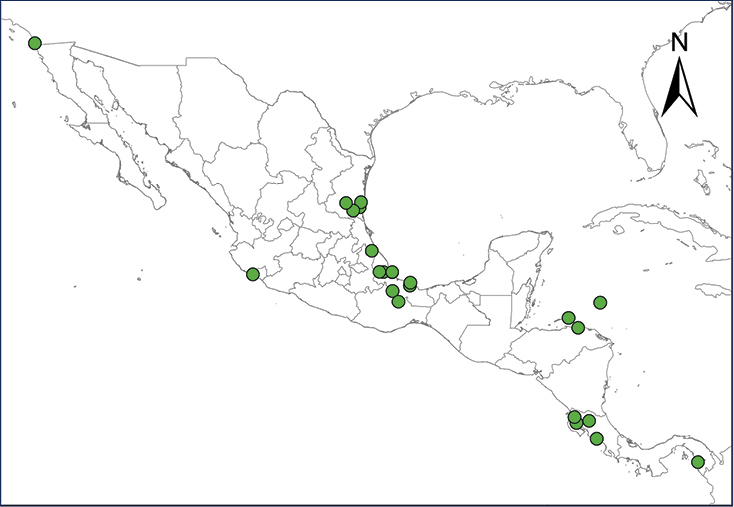	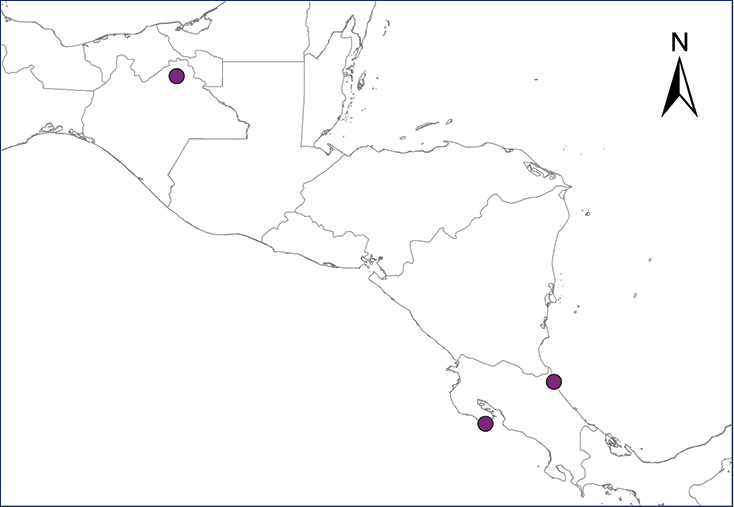
**Map 15.***Hemidactylus mabouia*.	**Map 16.***Hemidactylus garnotii*.
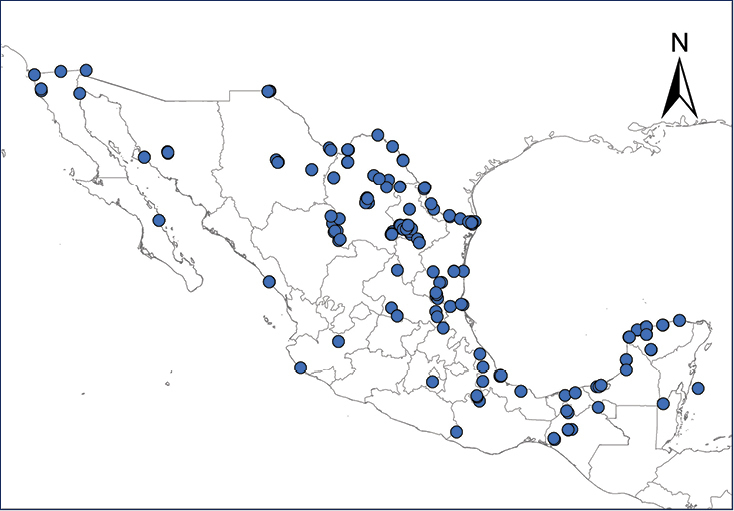	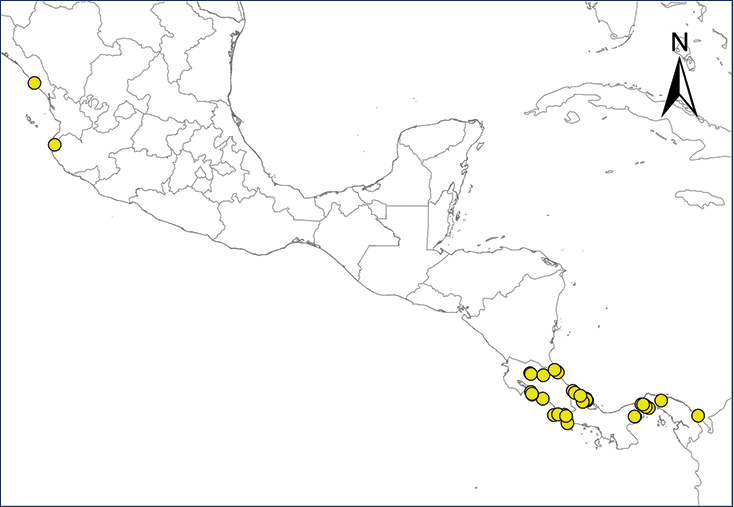
**Map 17.** *Hemidactylus turcicus*	**Map 18.** *Lepidodactylus lugubris*
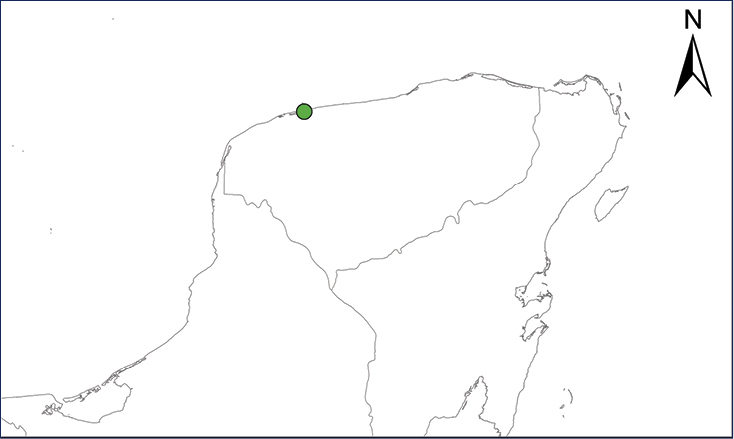	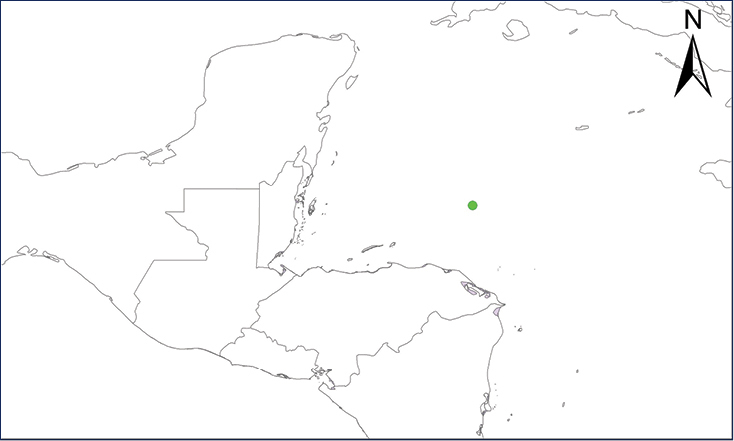
**Map 19.***Tarentola mauritanica*.	**Map 20.***Leiocephalus varius*.
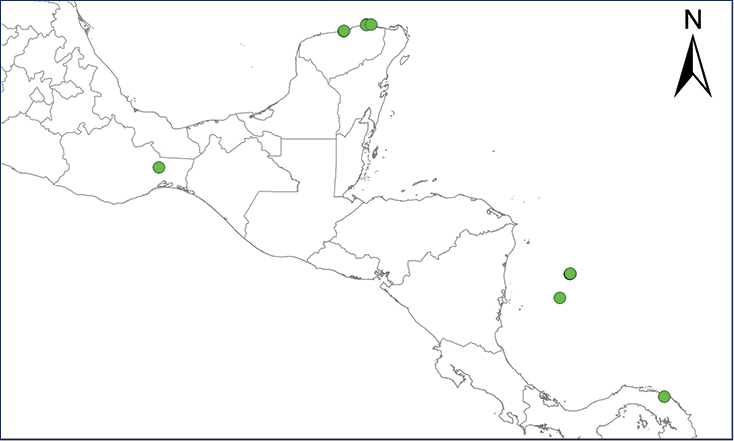	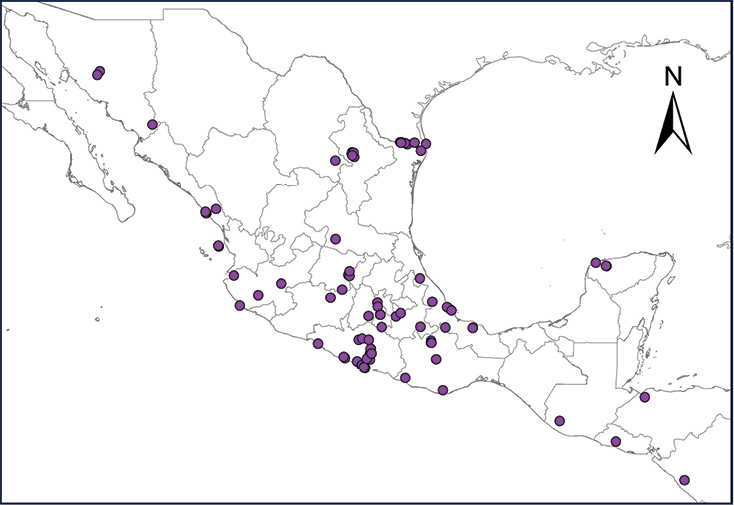
**Map 21.***Sphaerodactylus argus*.	**Map 22.***Indotyphlops braminus*.

***Eleutherodactylus coqui* (Thomas, 1966)**

Fig. 2

The Puerto Rican Coqui was first reported in Costa Rica by García-Rodríguez et al. (2010). According to them, it was a recent introduction, probably around the end of the 1990’s by a pet trader who carried six individuals from Puerto Rico and released them in Turrialba, where their descendants invaded surrounding localities despite attempts to exterminate them with poison; afterwards, survivors were sold as pets in other parts of Costa Rica. Besides Turrialba, however, the only other known established Costa Rican population is in nearby Juan Viñas (Barrantes-Madrigal 2017; Barrantes-Madrigal et al. 2019) (Table 2, Map 2). This is especially troubling since it has had extensive documented negative impacts on the native biota of Hawaii (Beard et al. 2002; Beard et al. 2009), and is listed as one of the 100 world’s worst invasive introduced species (Lowe et al. 2000). The control or eradication of this species is still possible, since its distribution apparently is still restricted to a few localities in Costa Rica (Barrantes-Madrigal 2017).

**Figure 2.**
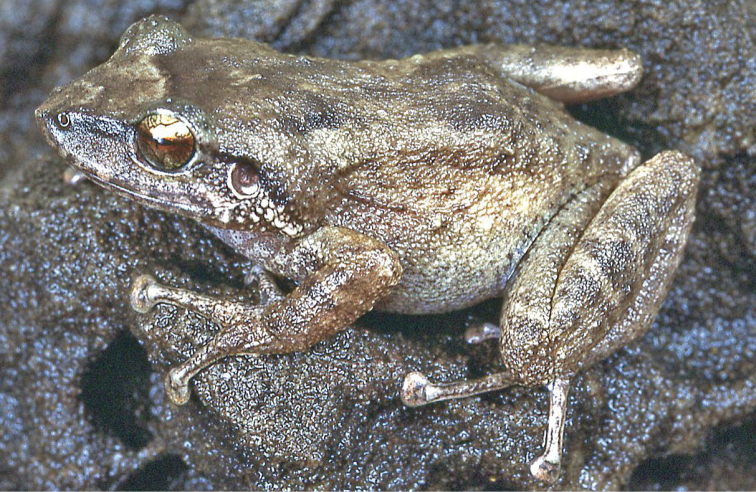
*Eleutherodactylus coqui*. Southern Florida. Photograph by Louis Porras.

***Eleutherodactylus johnstonei* (Barbour, 1914)**

Johnstone’s Whistling Frog is native to the Lesser Antilles and has been introduced into several Caribbean areas, South America, the United States, and Europe (Savage 2002). This frog is a particularly proficient invader since it easily establishes breeding sites and males vocalize almost immediately after being released into the wild, thereby out competing native frogs (Kaiser and Henderson 1994). In Central America, it currently occurs in residential suburbs of Panama City, where it was believed to have been introduced in the mid-1980’s, together with introduced plants (Ibañez and Rand 1990) (Table 2, Map 3). Savage (2002) reported a single specimen collected in a city park in San José, Costa Rica, but it apparently was alone and failed to establish a viable population (Savage and Bolaños-Vives 2005). Due to the lack of more records, Sasa et al. (2010) omitted this frog in their account of the Costa Rican herpetofauna. We agree with their opinion.

***Eleutherodactylus planirostris* (Cope, 1862)**

Fig. 3

The Greenhouse Frog is extremely small-sized (adults < 30 mm in length), is native to Cuba, Bahamas, and Cayman Islands (Crawford et al. 2011; Olson et al. 2012), and currently is found in Honduras in San Pedro Sula, La Paz, and Isla Guanaja, Islas de la Bahía (McCranie and Gutsche 2014). This frog has also been introduced into Puerto Limón, Costa Rica (Barquero and Araya 2016), the Miskito Cays of Nicaragua, in Panama, the Mexican Yucatan Peninsula (Cedeño-Vázquez et al. 2014; García-Balderas et al. 2016; Ortíz-Medina et al. 2017), and Veracruz, Mexico (Álvarez-Romero et al. 2008); the last authors noted that this species was first reported there in 1974; no other record existed from Veracruz until the phylogenetic analysis of Contreras-Calvario et al. (2018) concluded that the Greenhouse Frog population of the Gulf versant is related to Cuban populations, thus inferring a different colonization event from the *E. planirostris* on the Yucatan Peninsula, which is closer to Panama and Philippines populations (Cedeño-Vázquez et al. 2014). Recent reports, however, are found for Veracruz and Morelos in the iNaturalist platform (inaturalist.org 2018) (Table 2, Map 4). Environmental impacts produced by the diminutive Greenhouse Frogs need to be determined, since there has been no direct evidence for it being particularly harmful. Still, possible negative impacts include: predation on native invertebrates, competition for food with other insectivorous vertebrates, vulnerability to depredation that could limit its dispersal, but paradoxically it could become an abundant food source for other introduced species, thus facilitating their establishment and pending impacts (Olson et al. 2012).

**Figure 3.**
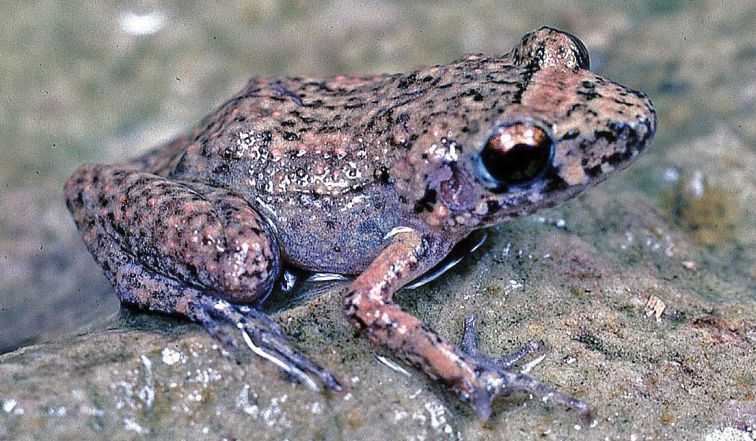
*Eleutherodactylus planirostris*. Southern Florida. Photograph by Louis Porras.

#### Family Hylidae

***Osteopilus septentrion,alis* (Duméril & Bibron, 1841)**

The Cuban Tree Frog is native to Cuba, Bahamas, and the Cayman Islands (Duellman 2001). In Central America, it is only known from Puerto Limón, Costa Rica, where it arrived in the mid 1980’s as an accidental introduction from cargo ships (Savage 2002) (Table 2, Map 5). Due to high adaptability to humid disturbed areas and its explosive breeding behavior, it has a high potential to spread quickly to other populated areas (Savage 2002).

#### Family Pipidae

***Xenopus laevis* (Daudin, 1802)**

Fig. 4

The African Clawed Frog is native to “extreme southern Angola…south to Cape Region of Rep. South Africa thence east and north in savanna habitats through Zimbabwe and southeastern Zambia to Malawi” (Frost 2020). This frog is now a widespread invader in lentic waters, and its accidental or deliberate introductions are associated with uses as a laboratory animal or pet (Álvarez-Romero et al. 2008; Measey et al. 2012). Although we know now that *Batrachochytrium* is native to Asia (O’Hanlon et al. 2018), the earliest known presence of *Batrachochytrium dendrobatidis* fungal infection outside its native range came from *X. laevis* populations in Africa (Weldon et al. 2004). Thus, the association with global trade markets and frequent releasing of this frog into the wild was credited for initiating the chytridiomycosis epidemics (Kraus 2009). The frog’s presence in Mexico was documented first in the 1980’s from a single observation (Murphy 1983), probably a result of dispersing individuals from established populations in southern California (Peralta-García et al. 2014). Today, it is confined to the Tijuana and Ensenada region in northwestern Baja California (Lavín et al. 2014), with large populations reported at sites near Rosarito (Peralta-García et al. 2014) (Table 2, Map 6). Further range expansion of this species is highly probable, since the landscapes all along northern Baja California and northern Mexican Plateau contain suitable habitats (Measey et al. 2012). It is not clear, however, if by “Baja” Measey et al. (2012) meant the total Baja California peninsula or only the Mexican state of Baja California.

**Figure 4.**
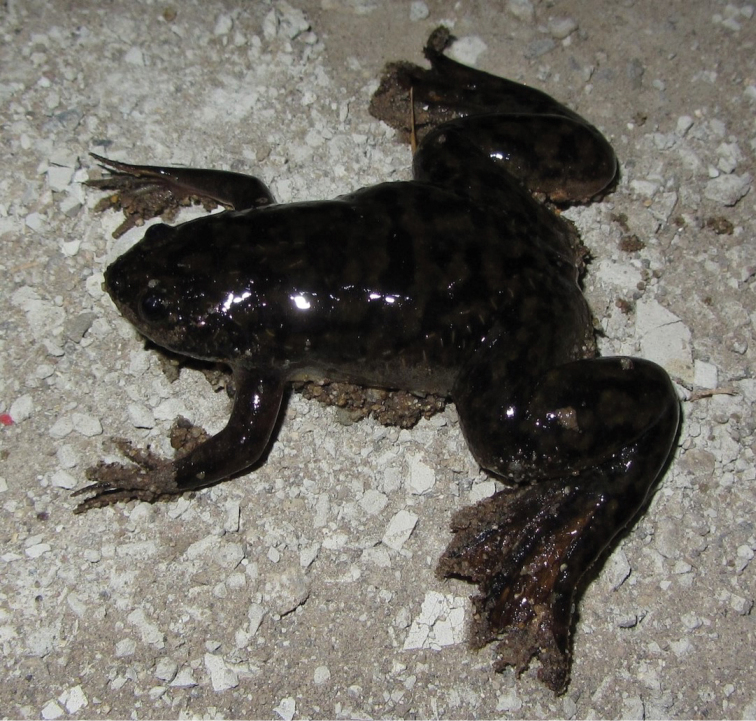
*Xenopus laevis*. Rosarito, Baja California, Mexico. Photograph by Anny Peralta.

### Reptiles – Testudines – Turtles

#### Family Chelydridae

***Chelydra serpentina* (Linnaeus, 1758)**

The Eastern Snapping Turtle is mentioned in the “Lista de las Especies Exóticas Invasoras para México” as present in the northern Mexican states of Coahuila and Chihuahua (SEMARNAT 2017), based on a distribution terminating at the Rio Grande as mapped by Stebbins (2003), who didn’t identify any verified records for Mexico. Several publications, including Lemos-Espinal and Smith (2007a, b), Lemos-Espinal and Smith (2016), Lemos-Espinal et al. (2017), and several chapters in Lemos-Espinal (2015) covering the US-Mexico border states alongside the Rio Grande, did not list *C. serpentina* as having substantiated records from Mexico. Terán-Juárez et al. (2016) discussed the probable presence of this species in the Río Grande basin across the border from Hidalgo County, Texas, and came to the conclusion that Eastern Snapping Turtles most likely inhabit the border region of south Texas and adjacent Mexico. Michael J. Forstner (personal communication) observed them in Tamaulipas, but didn’t take any voucher specimens. Dixon (2013) thought that the Hidalgo, Texas, and adjacent Tamaulipas turtles were introduced from allopatric native populations farther northeast in Texas, a pattern depicted on the map by Powell et al. (2016). Even though we do not have a map showing this species’ range in Mexico, we are under the impression that enough evidence now exists to consider Eastern Snapping Turtles in Tamaulipas as exotic in Mexico, being introduced originally from native Texas populations to the north.

Cupul-Magaña and Rubio-Delgado (2003) reported an Eastern Snapping Turtle from Puerto Vallarta, Jalisco, which they considered either a released individual or an escaped pet. Cruz-Sáenz et al. (2017) apparently did not think that the Puerto Vallarta individual was a member of an established population and did not list this species for Jalisco; we agree with that evaluation.

#### Family Testudinidae

***Chelonoidis carbonarius* (Spix, 1824)**

The Red-footed Tortoise´s original distribution range extends from central Panama, through Colombia and the Atlantic versant of the Amazonas in Brazil, as far south as Paraguay and northern Argentina (Köhler 2008). This species was listed originally as part of the Nicaraguan herpetofauna by Villa et al. (1988), who indicated that J. Villa found a single individual on Big Corn Island in 1964. They also mentioned the testimony of a local inhabitant who claimed to have owned a specimen of this species in her childhood, 50 years prior to the interview. If this testimony is true, the red-footed tortoise would have been on Big Corn Island by the end of the 1930’s. Thus, this tortoise was known on Great Corn Island by the second half of the 20^th^ century, but since the other known specimens were individuals held in captivity or kept as pets, the Red-footed Tortoise was omitted from the Nicaraguan herpetofauna by subsequent listings (e.g., Köhler 1999, 2001; Ruíz-Pérez 1996). Salazar-Saveedra et al. (2015), however, reported a breeding wild population on Great Corn Island, and pointed out that this population could be of exotic origin (Table 2, Map 7). They also stated that the known records of *C. carbonarius* on mainland Nicaragua (departments of Nueva Segovia and Masaya) might have originated from the Corn Islands. Sunyer and Martínez-Fonseca (2015) accepted the Red-footed tortoise as a member of the Nicaraguan herpetofauna and remarked that this species is alien in the country.

### Reptiles – Squamata – Lizards

#### Family Dactyloidae

The anole family Dactyloidae contains the second highest number of introduced species (four species) in Middle America, and has exotic members distributed in all countries except El Salvador and Nicaragua. *Norops sagrei* is the most widespread anole in Middle America.

***Anolis allisoni* (Barbour, 1928)**

Fig. 5

Allison’s Anole is listed by Álvarez-Romero et al. (2008) as being exotic in Mexico. Lee (1996) considered its occurrence on Cozumel Island doubtful, and González-Sánchez et al. (2017) reported that the only populations of this species in Mexico occur on the cays of Banco Chinchorro, Quintana Roo. *Anolis allisoni* also can be found on other Caribbean islands, such as Half Moon Cay, Belize (Schmidt 1941) and on Islas de Barbareta, Guanaja, Morat, Roatán, Utila, and Cayos Cochinos within the Islas de la Bahía complex in Honduras, as well as near the northern coastal regions, such as in La Ceiba, Atlántida (McCranie and Gutsche 2009), on the Honduran mainland; populations from Utila and La Ceiba might be recent introductions (McCranie and Köhler 2015) (Table 2, Map 8).

**Figure 5.**
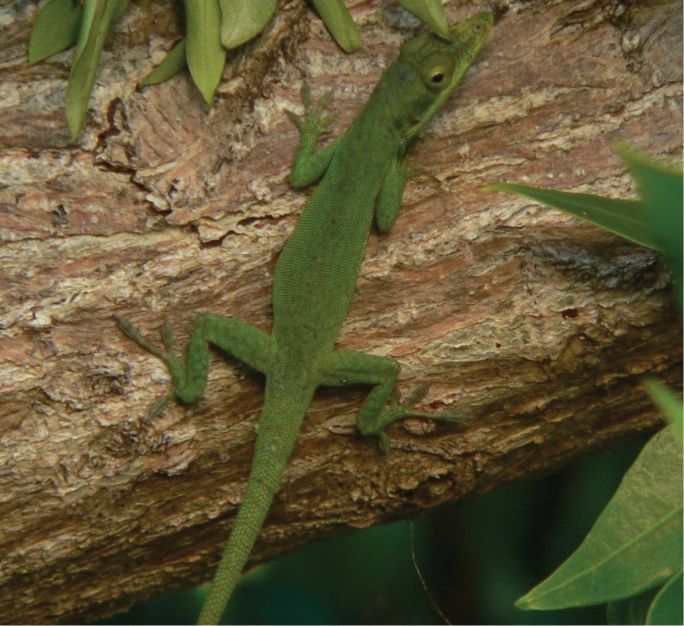
*Anolis allisoni*. Banco Chinchorro Atoll, Mexico. Photograph by Víctor H. González-Sánchez.

Recent evidence (Glor et al. 2005) indicated that *A. allisoni* is native to Cuba. The Mexican and Honduran populations have little variation in genetic distances when compared to Cuban populations, which suggests recent introductions onto the offshore islands of Mexico and Central America, as well as mainland areas of Honduras. In addition, McCranie and Gutsche (2009) indicated that the *A. allisoni* population (first mainland record for Honduras) in the Caribbean port of La Ceiba is a recent introduction from the Islas de la Bahía.

***Anolis carolinensis* (Voigt, 1832)**

Presence of the Green Anole in Mexico was suggested by Conant and Collins (1998), because of a supposed record from Tamaulipas, but no evidence was provided. Álvarez-Romero et al. (2008) advocated that at least one population in Tamaulipas might be native, but they did not provide any specific locality or give any justification for that reasoning. Farr (2015) did not list the Green Anole from Tamaulipas. The occurrence of this species in Tamaulipas remained controversial until Terán-Juárez et al. (2015) reported photographic records from a courtyard in Ciudad Valle Hermoso where *A. carolinensis* had been observed for at least ten years; they also indicated its presence in the city of Matamoros. We consider *A. carolinensis* an exotic species in Mexico, based on Dixon’s (2013) assertion that records from Texas border counties were probable introductions and that its known allopatric native distribution lies to the north of the border region in Texas (Powell et al. 2016; map only) (Table 2, Map 9).

In Belize, the Green Anole is only known from a single specimen collected in 1966 on Half-Moon Cay (UF 23924); no other records were reported thereafter (Lee 1996), although some lizards sighted in 1996 resembled *A. carolinensis* (Platt et al. 1999). This last claim should be treated with caution, as the very similar-looking *A. allisoni* also occurs on that cay, and can be easily misidentified. Lee (1996) hinted that if the Green Anole ever occurred on Half-Moon Cay, it might be extirpated now due to displacement by *A. allisoni*. Stafford et al. (2010) did not list *A. carolinensis* for Belize, so we adopt this view by not recognizing this species as presently having an established population in that country.

***Ctenonotus cristatellus* (Duméril & Bibron, 1837)**

Fig. 6

The Crested Anole is native to Puerto Rico and the Virgin Islands (Köhler 2008). In Central America, it has been established only in Costa Rica, where it has been observed in Puerto Limón (Savage 2002), Cahuita (Mayer 2010), Guayacán, and Valle de Rosas, Limón province, and in Turrialba, Cartago province (Savage 2002). This anole was first recorded in 1970 from Limón (Mayer 2010) and Fitch (1975) noted an explosive population increase of these anoles in a Limón city park and wondered if colonization by *C. cristatellus* might have caused the extirpation of *Gonatodes albogularis* in that park (Table 2, Map 10).

**Figure 6.**
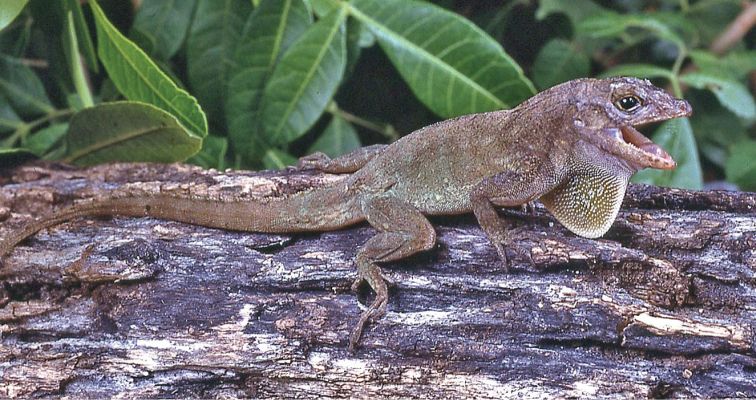
*Ctenonotus cristatellus*. Southern Florida. Photograph by Louis Porras.

In Mexico, *C. cristatellus* was reported from states on the Yucatan Peninsula, first by a single record from Cozumel, Quintana Roo, although Lee (1996) considered its presence there doubtful. Colston et al. (2015) reported *Norops cristatellus* from Calakmul, Campeche, but González-Sánchez et al. (2017) did not list it for the region because they thought the record needed verification, especially since Calakmul is a popular study site for working herpetologists and no other records are known from there. We concur that this species does not have an established population on Cozumel or Calakmul, so we remove it from the list of introduced species in Mexico.

***Norops sagrei* (Duméril & Bibron, 1837)**

The Cuban Brown Anole is native to Cuba, the Bahamas, and Cayman Islands, but it is unclear if all populations in Jamaica were introduced, or if some were native (McCranie and Köhler 2015). It is important to note that the taxonomic status and identity of this species remain unclear for many populations in Mexico and northern Central America, since the validity of the “native” subspecies *N. s. mayensis* seriously has been questioned (González-Sánchez et al. 2017). Moreover, the description of this subspecies was made from a single population from Isla Polao, in the region of Laguna de Términos, Campeche, and supported only by morphological characters (Smith and Burger 1949). Anoles as a group can have much geographic variability in morphology, as exemplified by McCranie and Köhler’s (2015: 169) statement, “using dewlap color as a diagnostic character for any *N. sagrei* complex population (including isolated island populations) might not be informative.” In any case, considering the unlikelihood that *N. s. mayensis* is a valid taxonomic lineage (see our taxonomic positions above in the methods section), and because of the long history of *N. sagrei* colonizing Mexico and Central America, any defining characteristics of a separate evolutionary lineage (=species) were probably eliminated by genetic intergradation with other alien *N. sagrei* populations. Because of this and the close association of this species with human mediated disturbed habitats, we regard the *N. sagrei* complex, with one exception, as a single exotic species within the region. The exception was the recent resurrection by McCranie and Köhler (2015) of *N. nelsoni*, an allopatric *N. sagrei* complex species from the Islas del Cisne, Honduras.

The naturalized distributional range in Mexico of *N. sagrei* comprises all the inland regions and several insular systems off the Yucatan Peninsula (González-Sánchez et al. 2017; Lee 1996), Tabasco (Lee 1996), Ciudad Altamira, Tamaulipas (Terán-Juárez et al. 2015), and Minatitlán and Catemaco, Veracruz (Zamora-Abrego et al. 2006). Specimens labeled *Anolis sagrei* in the Zoological Collection of El Colegio de la Frontera Sur (ECOSUR), in San Cristóbal de las Casas, Chiapas (Muñoz-Alonso 2006), are from Ocozocoautla de Espinosa (Coita), Chiapas, which is located in the semi-arid Central Depression region. Johnson et al. (2010, 2015a) did not report that locality and no other records are known to exist in Chiapas.

In Central America, the Cuban Brown Anole occurs throughout Belize (Lee 1996) and adjacent Caribbean lowlands of Guatemala (Stuart 1955). In Honduras, this species is known to occur at Puerto Cortés and San Pedro Sula, Cortés, and at La Ceiba and Tela, Atlántida, on the northern mainland and on the islands of Roatán and Utila (McCranie and Köhler 2015). An introduced population also exists in the vicinity of Limón, Costa Rica (Savage and Bolaños-Vives 2005). Batista et al. (2019) reported established populations of *N. sagrei* at several sites within and around Panama City. They also mentioned that those populations might have become established approximately five years ago, and arrived there as released pets, or more probably, from shipments arriving at the port of Balboa (Table 2, Map 11).

#### Family Gekkonidae

Not surprisingly, the geckos contain the highest number of introduced species (10 species) for the region, distributed among two families (i.e., Gekkonidae and Sphaerodactylidae) in Middle America (Table 1). Because of their notorious colonization ability, geckos are frequently referred to as “weedy” species (Kluge 1969).

*Hemidactylus* is the most speciose genus, comprising five introduced species (*H. frenatus*, *H. garnotii*, *H. haitianus*, *H. mabouia*, and *H. turcicus*). At least one of these species occurs in each country of Middle America, but only *H. frenatus* is present in all seven (Table 2). *Hemidactylus* is a species-rich genus (167 species; Uetz et al. 2020), native to tropical areas of Asia and Africa and the Mediterranean region, most of which species have small native distributional ranges. A few species, however, can be found almost worldwide in tropical and subtropical areas due to either human intervention or possibly by having undertaken long transmarine journeys (Carranza and Arnold 2006).

***Gekko gecko* (Linnaeus, 1758)**

Fig. 7

The Tokay Gecko is very well known due to its use in scientific research (Roesler et al. 2011), and popularity in the pet trade (Kraus 2009). It was originally native to eastern India, Nepal, Southeast Asia, China, and the Malayan Archipelago (Roesler et al. 2011). Stafford et al. (2010) listed this species as exotic in Belize. The population was reported from South Water Caye, a small sandy caye measuring 8.2 ha on the Belizean barrier reef (Meerman and Garel 2002) (Table 2, Map 12). Apparently, the introduction of the Tokay Gecko occurred around 1994, when a tourism industry worker brought several individuals to South Water Caye and intentionally released them. The introduction of *G. gecko* and the declining numbers of *Aristelliger georgeenis* and *Phyllodactylus tuberculosus* on that island might be related (Meerman and Garel 2002).

**Figure 7.**
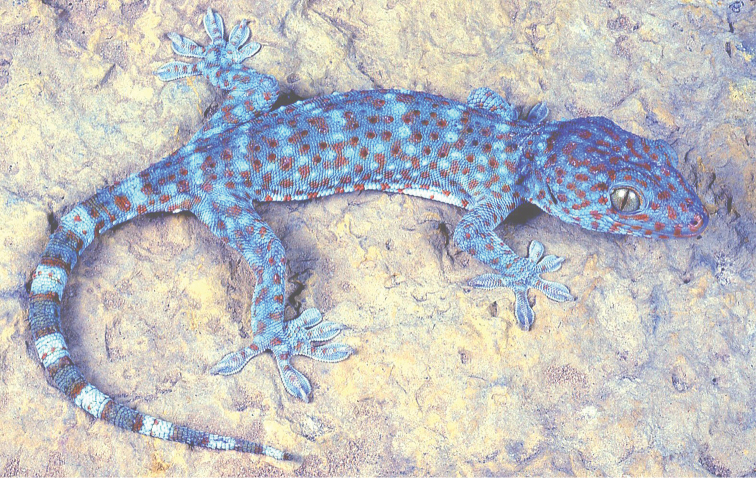
*Gekko gecko*. Southern Florida. Photograph by Louis Porras.

***Gehyra mutilata* (Wiegmann, 1834)**

The Stump-toed Gecko is native to the Pacific basin region of Southeast Asia, where it has dispersed among Indian and Oceanic islands since the times of pre-Polynesian navigators (Fisher 1997). Rocha et al. (2009) suggested that *G. mutilata* is a complex of two cryptic lineages, one of them involving the resurrection of *G. insulensis* for several, if not all, Pacific islands, which includes the former Mexican, but now French Isla Clipperton (Isla de la Pasión). Lorvelec and Pascal (2006) hypothesized that the time and source of invasion on that atoll could have been during the 1950’s from Mexican Pacific ports in Nayarit or Sinaloa, but that allegation was merely speculation. If true, however, Mexican populations on the Pacific side would correspond to *G. insulensis*. Since Rocha et al. (2009) did not include Mexican samples in their study, we retain the name *G. mutilata* until more evidence is provided.

The date for the introduction of *G. mutilata* into Mexico is unknown, although Lemos-Espinal and Dixon (2013) remarked that before the advent of aviation, this species was restricted almost exclusively to the Philippines, although specimens collected in Nayarit by the end of the 19^th^ century already existed (GBIF.org 2018). In addition, Ineich and Blanc (1987) hypothesized that it could have been present in Mexico during the 18^th^ Century by way of maritime trade between New Spain and the Philippines. In Mexico, it is now known from the Pacific versant in Sinaloa, Nayarit, Guerrero, and Chiapas (Álvarez-Romero et al. 2008), Jalisco (Cruz-Sáenz et al. 2017), and Baja California Sur (Reynoso 1990a; Lovich et al. 2009), and some Pacific Islands, such as Isabel (Quijada-Mascareñas and Canseco-Márquez 2007). On the Atlantic versant, established populations have been reported only from Veracruz (Álvarez-Romero et al. 2008) and Ciudad Valles, San Luis Potosí (Lemos-Espinal and Dixon 2013) (Table 2, Map 13).

***Hemidactylus frenatus* (Duméril & Bibron, 1836)**

Fig. 8

The Common House Gecko is a well-known successful colonizer of urban environments (Lee 1996). Even though its native range is uncertain, it probably can be restricted to southern India, Sri Lanka, Burma, southern China, Malayan Peninsula, and Philippines (Savage 2002). As an introduction in the Americas, it occurs on both versants from Florida and California through Mexico and Central America to Brazil (Weterings and Vetter 2017), from sea level to 1,545 m elevation (Mata-Silva et al. 2013). Introductions of this gecko possibly could lead to competitive exclusion of native gecko populations and to extinction of insular endemics (Cole et al. 2005).

**Figure 8.**
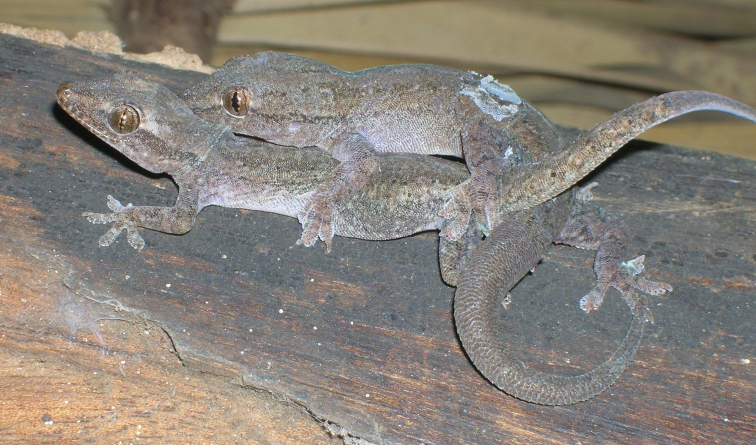
*Hemidactylus frenatus*. León, Nicaragua. Photograph by Javier Sunyer.

It is believed that *H. frenatus* arrived on the North American continent around the 16^th^ century by means of maritime commerce between the Philippines and Acapulco, Mexico (Álvarez-Romero et al. 2008). Farr (2011), however, argued that the first records of this species date to the end of the 19^th^ century or even as late as the 1930’s. If so, colonization during the colonial period, by an introduced lizard common in hotels of Acapulco today, should have been recorded early on by the first collectors visiting Mexico, but since there are no records from then, introductions into Central American countries might have been even more recent. For example, in Costa Rica the first reports of *H. frenatus* were made after 1990, according to Savage (2002). This exotic species also has been reported from all other Central American countries and many states in Mexico (Uetz et al. 2020). *Hemidactylus frenatus* first was recorded from peninsular Baja California Sur, Mexico, by Reynoso (1990b) from the city of La Paz; Grismer (2002) also observed an established population in Loreto, located north of La Paz on the peninsula. Most recently, the species was reported for the first time from any island in the Sea of Cortes (Isla El Pardito) by Dayton et al. (2020) (Table 2, Map 14). Mata-Silva et al. (2013) found this species to be very common in Oaxaca City, Oaxaca at an elevation of 1,545 m.

***Hemidactylus garnotii* (Duméril & Bibron, 1836)**

The Indo-Pacific or Garnot’s House Gecko, is native to the Indo-Pacific basin, and is widespread on several islands in Oceania, Asia, and the Pacific Ocean. In Middle America, it was introduced at several ports and urban centers in Costa Rica (Savage 2002; Köhler 2008) and in Guatemala, Guatemala (Morales et al. 2017). This species is a parthenogenetic all-female species and, therefore, it seems to have no social hierarchy (Frankenberg 1982); it is a territorial and aggressive species (Frankenberg 1984), and successful colonizer of urban and other anthropogenic habitats, with high potential for expansion (Savage 2002). The first record in Middle America was from San José, Costa Rica, in 1992 (Savage 2002), whereas in Guatemala its introduction seems to be more recent, but from an unknown source (Morales et al. 2017) (Table 2, Map 16).

***Hemidactylus haitianus* (Meerwarth, 1901)**

The Haitian House Gecko has had a complex and unclear taxonomic history. Traditionally, it was considered two different taxa: the Middle American and West Indian populations of *H. brooki haitianus*, type locality marked as “Haití, Port-au-Prince” (Powell and Maxey 1990), and *H. angulatus*, type locality “West Coast of Africa” (Hallowell 1854). Powell et al. (1996) considered *H. brooki haitianus* a full species (*H. haitianus*) for lizards native to the West Indies, including Cuba and Puerto Rico. Further revisions revealed *H. haitianus* as nested inside a larger clade that includes the African populations of *H. brooki* along with *H. angulatus* (Carranza and Arnold 2006; Weiss and Hedges 2007; Bauer et al. 2010; Rösler and Glaw 2010). Weiss and Hedges (2007) and Gamble et al. (2011) reported virtually no genetic divergence among the populations of this complex in the Greater Antilles, and clustered them with African populations, thus corroborating the introductory origin of *H. haitianus* for the Antilles. The source of invasion to the New World might have been through the slave trade between western Africa and the West Indies (Weiss and Hedges 2007). Consequently, the populations of *H. brooki* in Honduras (McCranie 2015) and Panama (Auth 1994) and those of *H. angulatus* in South America might have had their origins by following colonial trade routes between the Antilles and Middle America, although a date has not been suggested. Even if the *H. angulatus-brooki* complex is not fully resolved, the name *H. haitianus*, as suggested by Bauer et al. (2010), is used herein for individuals occurring throughout the Western Hemisphere.

***Hemidactylus mabouia* (Moreau De Jonnès, 1818)**

Moreau’s Tropical House Gecko has no clear-cut geographic origin. The type locality is marked as “Antilles,” and restricted to St. Vincent Island by Stejneger (1904). Nonetheless, its actual origin was without doubt on the African continent, where it is widespread, ranging from southern Africa northward to Liberia and Ethiopia (Álvarez-Romero et al. 2008). An interesting hypothesis suggests arrival of this species in the New World by accidental transport on slave ships along routes from Africa to the West Indies and South America. There is no full concordance, however, between slave ship routes and the distribution of *H. mabouia* (Kluge 1969). Based on the long history of maritime trade between Africa and the Antilles and the 1818 description of the species, it can surely be said that the type specimen from the Antilles represents an introduced population. Moreover, the introductions in Mexico and Central America might be the result of maritime trade from the West Indies (Álvarez-Romero et al. 2008). Currently, introduced populations of *H. mabouia* are found in Veracruz (Ochoa-Ochoa et al. 2006; Álvarez-Romero et al. 2008), and Tamaulipas, Mexico (Sosa-Tovar, et al. 2019), Islas de la Bahía, Honduras (Gutsche and McCranie 2009), San José, Costa Rica (Abarca and Monge 2007), and Panama (Auth 1994) (Table 2, Map 15).

***Hemidactylus turcicus* (Linnaeus, 1758)**

The Mediterranean House Gecko is native to coastal areas of the Mediterranean, where it is widespread across southern Europe, the Levant, and more sporadically in North Africa (Martínez-Hernández et al. 2017). In Mexico, it is known from many states and places, including: Yucatan Peninsula, Baja California, Sonora, Sinaloa, Chihuahua, Coahuila, Durango, San Luis Potosí, Aguascalientes, Ciudad de Mexico, Morelos, Nuevo León, Puebla, Tamaulipas, Oaxaca, Chiapas (Martínez-Hernández et al. 2017), and Querétaro (Tepos-Ramírez, 2019). Its first introduction probably occurred around Acapulco, from colonial-period trade with inhabitants of the Pacific islands (Álvarez-Romero et al. 2008), although another possible source was from Veracruz, where *H. turcicus* was recorded in 1895. Subsequently, *H. exsul* was described in 1906 from Progreso, Yucatán, which in fact was based on a specimen of *H. turcicus* (McCoy 1970). In Panama, it is known from the Canal Zone (McCoy 1970), where the introduction took place after the opening of the Panama Canal in 1914. Stafford et al. (2010) suggested the possible presence of this species in Belize, but we were unable to find records on GBIF.org (2018), or in the available literature, so it is not recognized herein for that country (Table 2, Map 17).

***Lepidodactylus lugubris* (Duméril & Bibron, 1836)**

Fig. 9

The Mourning Gecko is native to southeast Asian and Indo-Australian regions, and currently is distributed worldwide in the tropics from sea level up to 700 m elevation (Köhler 2008). Probably much of its dispersal potential comes from being a complex of parthenogenetic lineages that includes diploid and triploid forms because of hybridization between *L. moestus* and an undescribed species (Fujita and Moritz 2009). In the Americas, it was first reported in the mid 1950’s at Ft. Clayton, in the Canal Zone, Panama (Smith and Grant 1961). Even though prior collected specimens exist from the 1910’s in that area, it is also known from Bocas del Toro, Panamá (Hoogmoed and Avila-Pires 2015), Golfo Dulce, and Peninsula de Osa and Punta Arenas departments, both from the Pacific versant of Costa Rica (Savage 2002). This species is known also in Costa Rica from the Gandoca-Manzanillo Wildlife Refuge, Talamanca, Limón, and from Tirimbina Biological Reserve and La Virgen of Sarapiquí, Heredia (Jiménez and Abarca 2014). This gecko is established in Nicaragua on the southeastern Caribbean coast at elevations lower than 10 m (Hoogmoed and Avila-Pires 2015), and on the Corn Islands (Sunyer et al. 2013), where individuals probably arrived on cargo ships sometime around 1975 at Bluefields and/or Great Corn Island (Henderson et al. 1976). Hoogmoed and Avila-Pires (2015) thought that the Mourning Gecko should not be listed for Mexico because of inconsistences in the literature and lack of vouchered museum specimens. A recent (November 2017) photographic record for the port of Mazatlán, Sinaloa, however, exists in the iNaturalist platform (GBIF.org 2018). In addition, Ahumada-Carrillo and Weatherman (2018) found a recent established population in Puerto Vallarta, Jalisco, Mexico (Table 2, Map 18).

**Figure 9.**
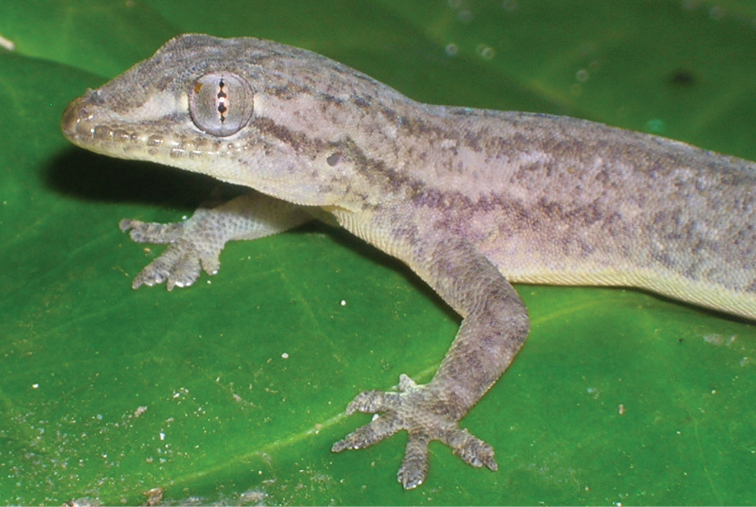
*Lepidodactylus lugubris*. Big Corn Island, Nicaragua. Photograph by Javier Sunyer.

***Tarentola mauritanica* (Linnaeus, 1758)**

Fig. 10

The Moorish Gecko is native to the European and North African Mediterranean basin (Rato et al. 2015). Ortíz-Medina et al. (2019) recently reported this species from cargo containers stored in a warehouse in Progreso, Yucatan, Mexico. They mentioned that six to eight “unusual-looking lizards” were sighted there in early 2017, but only two specimens were captured and identified as *T. mauritanica*. The population in Progresso, however, was supposedly established, which is most likely factual, considering the number of individuals observed at the site, in addition to the well-known success of gekkonid lizards for becoming established species. Another record is known from Guadalajara, Mexico, through a photographic entry in inaturalist.org (2018), but no other records of the Moorish Gecko exist for Mexico, and no validating museum vouchers could be found for Guadalajara (Table 2, Map 19). We have not overlooked the recent evidence suggesting that *T. mauritanica* constitutes a species complex (Rato et al. 2016), but choose to maintain the traditional nomenclature while awaiting full resolution of the group.

**Figure 10.**
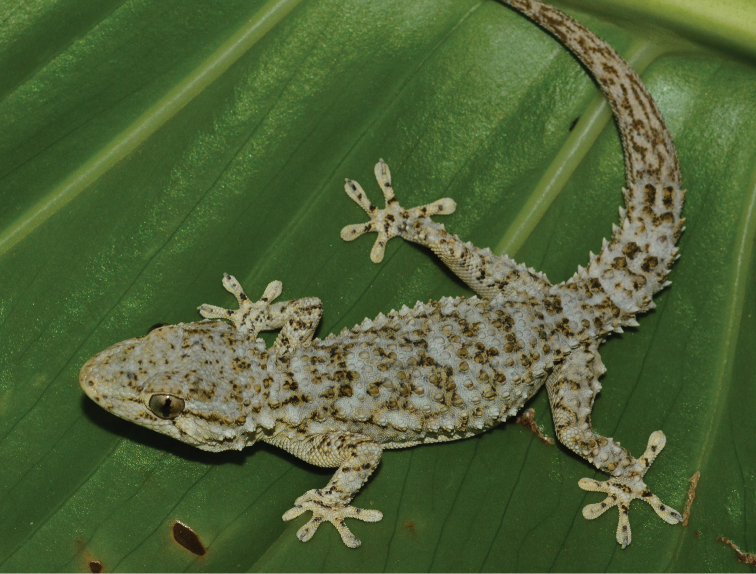
*Tarentola mauritanica*. Progreso, Yucatan, Mexico. Photograph by Javier Ortíz-Medina.

#### Family Leiocephalidae:

***Leiocephalus varius* (Garman, 1887)**

The Cayman Curly-tailed Lizard is native to the Grand Cayman Islands (Garman 1887; Echternacht et al. 2011) and was first reported on the Swan Islands, Honduras, in the mid 1970’s (Schwartz and Thomas 1975), where it was conspicuous on human buildings and other places on Big Swan Island (McCranie et al. 2017). While some authors listed it as native to Honduras (Townsend and Wilson 2010; Solís et al. 2014), McCranie et al. (2017) suggested that this lizard was introduced recently to the Swan Islands, although circumstances or the date of the introduction were not provided. We note that McCranie (2018) recently elevated *L. varius* to a full species from *L. carinatus varius*, therefore, *L. varius* replaces *L. carinatus* as the exotic species on the Swan Islands (Table 2, Map 20).

#### Family Sphaerodactylidae

***Sphaerodactylus argus* (Gosse, 1850)**

The Ocellated Dwarf Gecko is native to Jamaica, Cuba, and adjacent islands in the Bahamas and Antilles, including the Colombian Isla de San Andrés (Harris and Kluge 1984). It was introduced into the northern Yucatan Peninsula of Mexico (Lee 1996), Isla del Maíz Grande (Corn islands), Nicaragua (Thomas 1975; Sunyer et al. 2013), and on several islands on the Bocas del Toro and San Blas Archipelagos in Panama (Harris and Kluge 1984; Savage 2002). Approximate dates of introductions on the islands of Central America are unknown, but before Thomas (1975) no previous record for Middle America was available.

Records of *S. argus* from the northern coast of Yucatan (Lee 1996) might be the result of active maritime commerce in the region. Furthermore, this gecko was mentioned as occurring on the insular systems of Costa Rica (Lee 1996), but no evidence or citation was provided. *Sphaerodactylus argus* has not been reported on mainland Costa Rica (Savage 2002; Savage and Bolaños-Vives 2005; Köhler 2008; Sasa et al. 2010), although its close proximity to the Bocas del Toro islands could lead to future colonization of mainland areas adjacent to those islands (Savage 2002). *Sphaerodactylus argus* should not be confused with what was formerly known as *S. argus continentalis* (now *S. continentalis*), which ranges from the Isthmus of Tehuantepec, Mexico, into central Honduras (McCranie and Hedges 2012) (Table 2, Map 21).

### Reptiles – Squamata – Snakes

#### Family Typhlopidae

***Indotyphlops braminus* (Daudin, 1803)**

Fig. 11

The Brahminy Blindsnake is the most widespread alien reptile in the world (Capinha et al. 2017). This snake is a small-sized (mean total length < 130 mm) brown to black-colored species (Wallach 2009), with a secretive fossorial lifestyle occupying soil and leaf litter (Álvarez-Romero et al. 2008), and can easily be confused with earthworms (Wallach 2009). The wide distributional range of this snake can be explained by the ease with which this snake is carried inadvertently within root masses of potted plants being shipped world-wide by the garden industry (Álvarez-Romero et al. 2008). The reproductive characteristics of this species (unisexual, triploid, and parthenogenetic) also allows a single individual to establish a new population (Vitt and Caldwell 2014).

**Figure 11.**
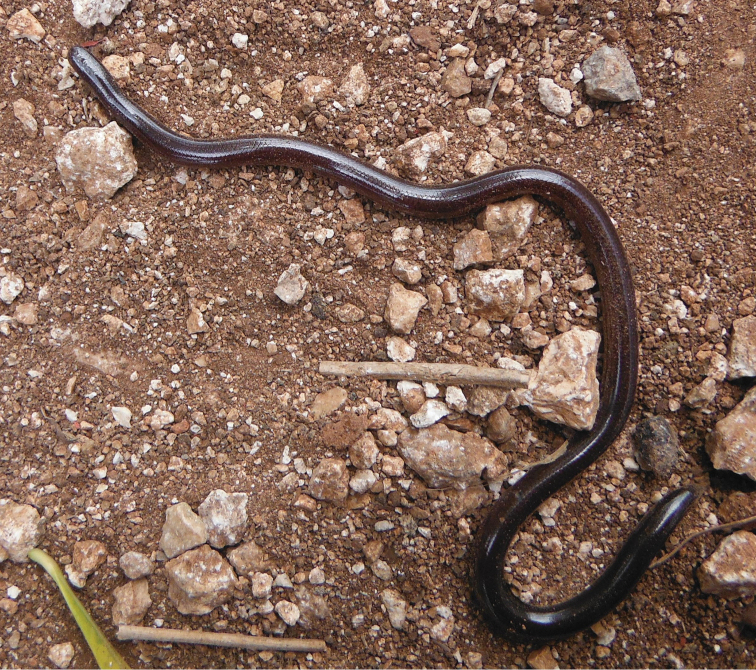
*Indotyphlops braminus*. Ixil, Yucatan, Mexico. Photograph by Luis Díaz-Gamboa.

The type locality is reportedly the region of Coromandel, southeast India (Wallach et al. 2014). The center of origin of *I. braminus* is difficult to discern with precision, however, due to its almost cosmopolitan distribution (Wallach et al. 2014; Capinha et al. 2017), previous unclear taxonomy, frequent misidentifications (Wallach 2009), and a long history of exceptional dispersal ability. The only certain location for the center of origin is that it should be an undetermined site in the Old-World tropics (Álvarez-Romero et al. 2008). This snake also has a hybrid origin with the parental species still undetermined (Vitt and Caldwell 2014). Currently, it is widespread in Mexico, Guatemala, Belize, Honduras, and El Salvador (Wallach et al. 2014; Lee 1996). In Nicaragua, it is known from a recent record (Leets-Rodríguez et al. 2019) from a locality near Managua. Due the nature of the record (five specimens found accidentally in a suburban backyard), it is probable that the Brahminy Blindsnake is widespread in urban and suburban areas of Managua. Arrival in Mexico was most likely sometime during the 16^th^ century via maritime trade between the Philippines and New Spain at the Acapulco port (Álvarez-Romero et al. 2008). Care should be taken not to confuse *I. braminus* with other local members of Typhlopidae inhabiting the American tropics (Table 2, Map 22).

### The translocated herpetofauna of Middle America

Several species of amphibians and reptiles found in a region are translocated when introduced into areas of the same region outside their native ranges. We will consider only those with sound evidence of having been translocated by human activities and that have established populations (Table 1).

### Amphibia – Anura – Frogs

#### Family Hylidae

***Smilisca baudinii* (Duméril & Bibron, 1841)**

The Mexican Treefrog ranges from “Extreme southern Texas (USA) and southern Sonora and southwestern Chihuahua (Mexico) south (including the Balsas Depression of Mexico) in tropical lowlands to Costa Rica on the Pacific slope; including the Tres Marias Islands off the coast of Nayarit, Mexico” (Frost, 2020). This species also can be found on other Mexican Pacific Islands (Woolrich-Piña et al. 2016), but historically was unknown on the Baja California Peninsula (Grismer 2002; Lovich et al. 2009). Records of *S. baudinii* exist from near the village of Todos Santos, Baja California Sur, which could represent an unlikely relictual population, or a more reasonably explained accidental translocation from any of the mainland ferry ports where this frog naturally occurs (Recuero et al. 2004) (Table 3, Map 23).

**Table 3.** Non-native distribution of translocated amphibians and reptiles in Mexico and Central America.

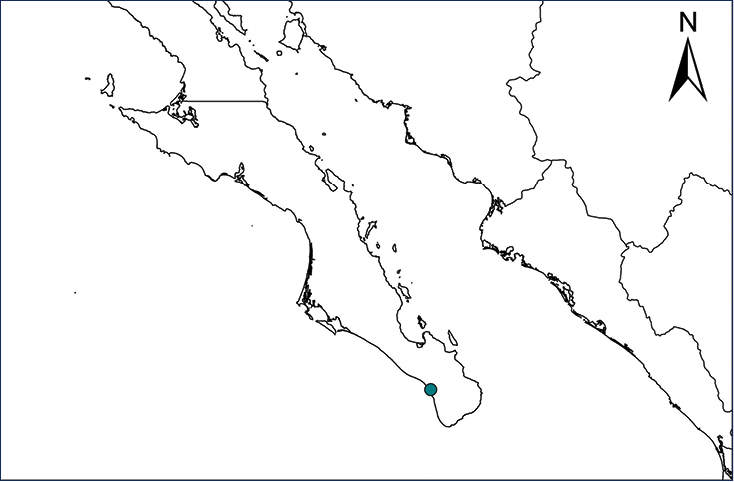	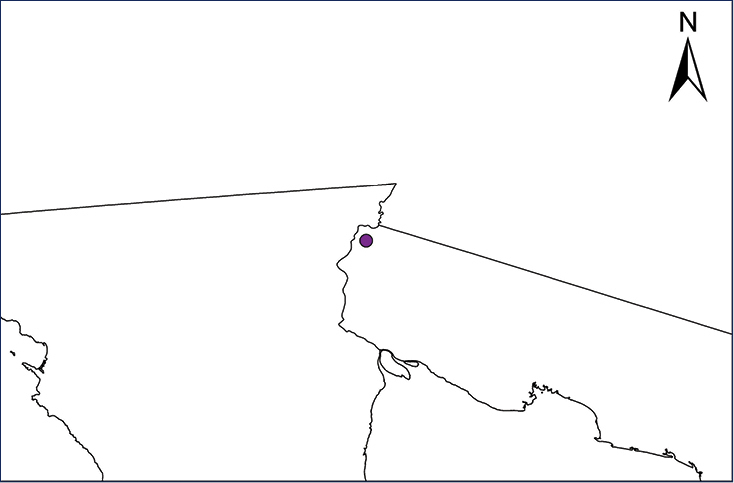
**Map 23.** *Smilisca baudinii*	**Map 24.** *Lithobates berlandieri*
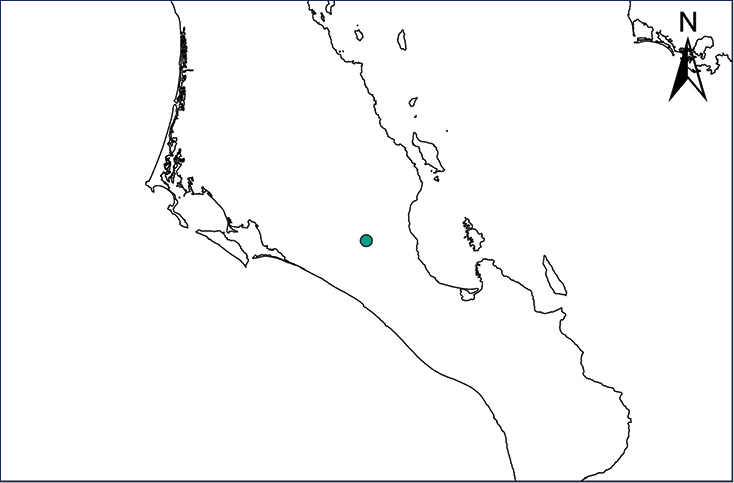	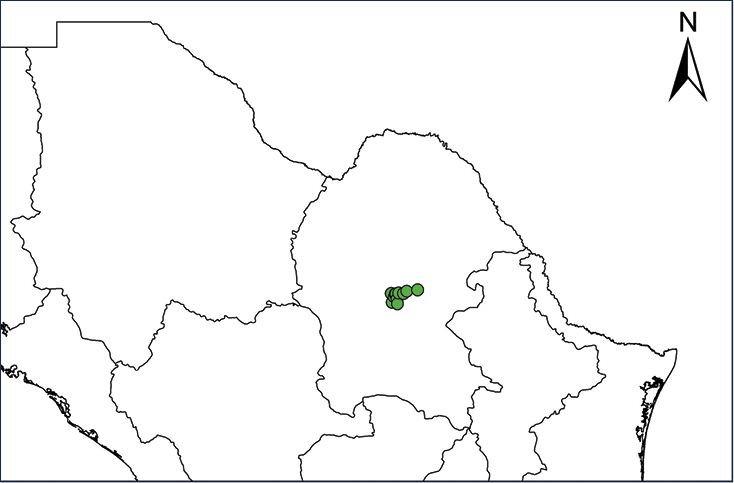
**Map 25.** *Lithobates forreri*	**Map 26.** *Apalone spinifera*
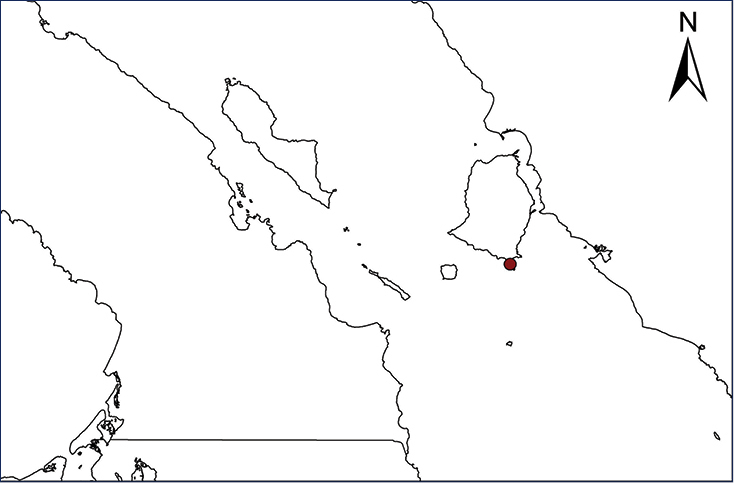	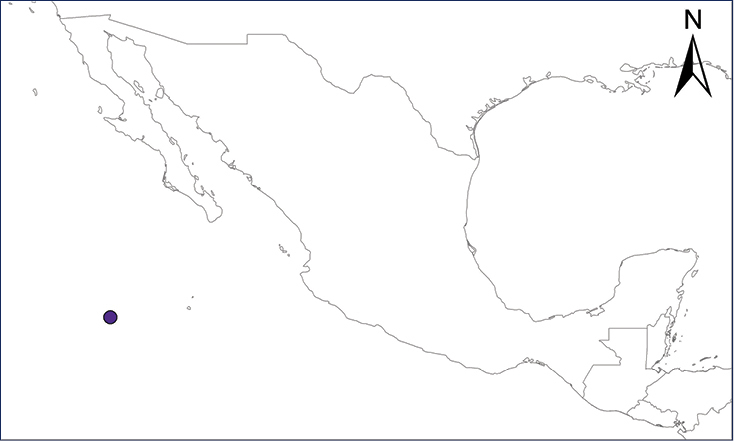
**Map 27.** *Ctenosaura conspicuosa*	**Map 28.** *Ctenosaura pectinata*
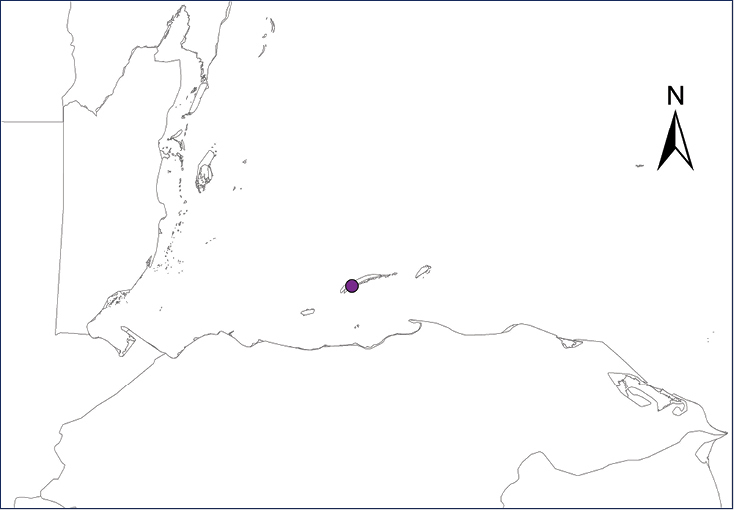	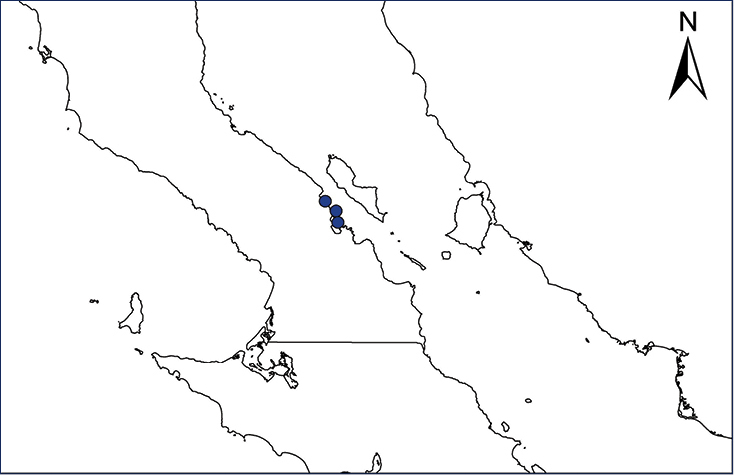
**Map 29.** *Ctenosaura similis*	**Map 30.** *Sauromalus hispidus*
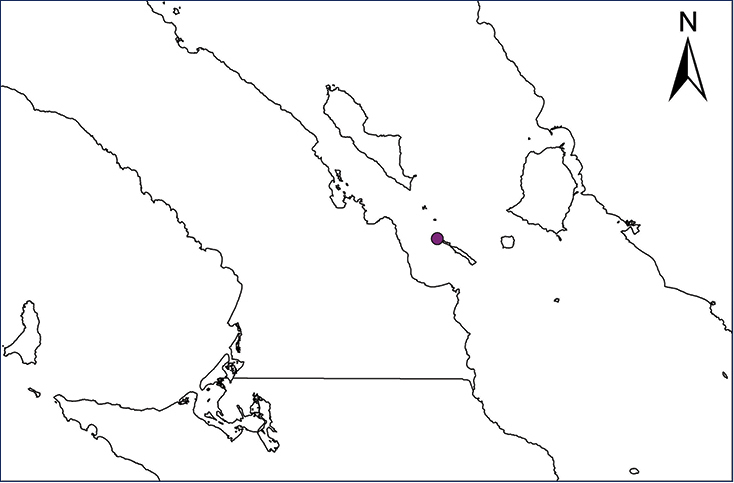	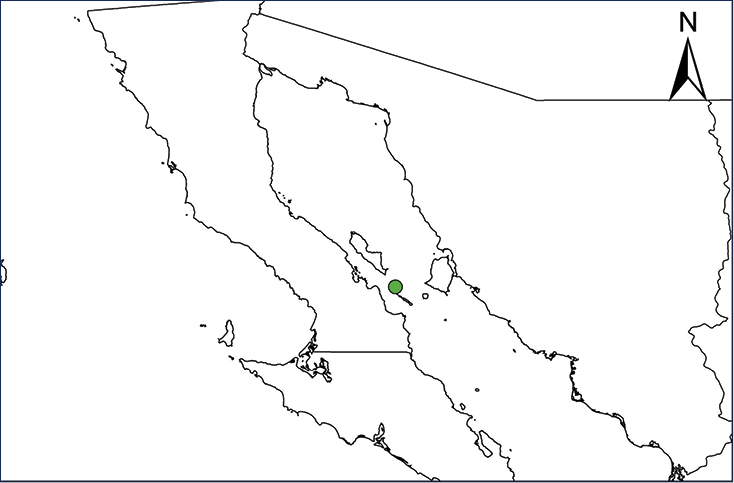
**Map 31.** *Sauromalus varius*	**Map 32.** *Uta stansburiana*
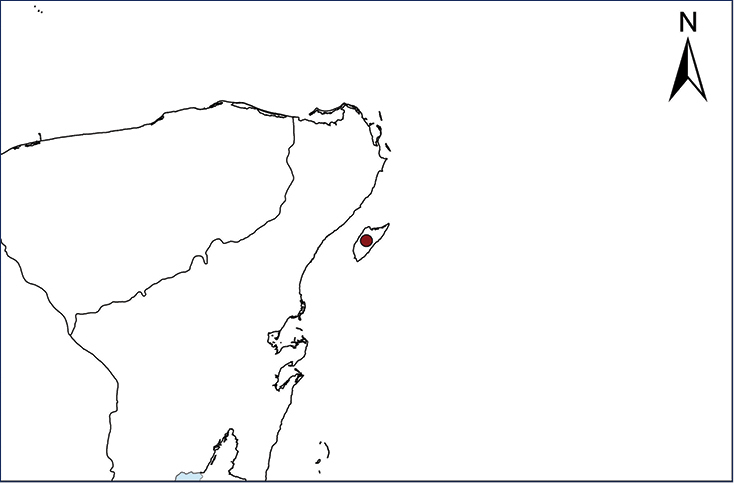	
**Map 33.** *Boa imperator*	

#### Family Ranidae

***Lithobates berlandieri* (Baird, 1859)**

The native range of the Rio Grande Leopard Frog extends from southeastern New Mexico and central Texas southward into Mexico (Stebbins 2003) along the Gulf lowlands into the northern half of Veracruz (Zaldívar-Riverón et al. 2004). It is unclear what the status is for populations in the Mexican portion of the lower Colorado Basin, where Grismer (2002) reported a sighting of what he believed to be an individual *L. berlandieri* at the confluence of the Hardy and Colorado rivers in the Mexicali Valley, Baja California. Photographic evidence of this species near San Luis Río Colorado, Sonora, Mexico, was reported by Rorabaugh and Servoss (2006) from a concrete-lined ditch passing through an agricultural field. The invasion front originated somewhere in southwestern Arizona where the Colorado and Gila rivers meet, which is the same area where *L. berlandieri* was collected in a survey in 1981 (Platz et al. 1990). The most probable act of introduction into Mexico was not a single event, but rather a secondary effect of several fish transplant operations into the Yuma, Arizona region from New Mexico in the late 1960’s or early 1970’s (Platz et al. 1990). It is likely that the Mexican populations, if they are established, originated as an expansion of the invasion front, using the Colorado and Gila River systems, and adjacent agricultural canals as dispersal routes (Rorabaugh et al. 2002). It is unknown what the impact of *L. berlandieri* is on the biodiversity of Baja California, but it has been associated with historical declines of populations of other native leopard frogs, such *L. yavapaiensis*, in areas of the lower Rio Colorado basin in Arizona (Rorabaugh et al. 2002) (Table 3, Map 24).

***Lithobates catesbeianus* (Shaw, 1802)**

Fig. 12

The American Bullfrog originally ranged from southeastern Canada and central and eastern United States into northeastern Mexico (Conant and Collins 1998). It has a long and extensive history of introductions into Mexico, with first reports made near Cadereyta, Nuevo León, in 1853 (Ramos-Guerra and Gatica 2014). Since then, feral populations have been established in many Mexican states and places, including Chihuahua, Durango, and San Luis Potosí (Lemos-Espinal and Smith 2016), Sinaloa, Sonora, Morelos, Ciudad de Mexico, Puebla, San Luis Potosí (Casas-Andreu et al. 2001), Aguascalientes (Avila-Villegas et al. 2007), Baja California (Grismer 2002) and Hidalgo (Ramírez-Bautista et al. 2014), where it is probably linked to the extirpation of *L. yavapaiensis* and *Incilius alvarius*, and declines in *Hyliola cadaverina* (as *Hyla regilla*) and *Thamnophis hammondii* in several oases on the Baja California Peninsula (Grismer 2002). Apparently, American Bullfrogs were introduced intentionally in Costa Rica (Savage and Bolaños-Vives 2005), although the previously known population in La Garita is now thought to be nonexistent (IUCN 2015) (Table 3, Map 25).

**Figure 12.**
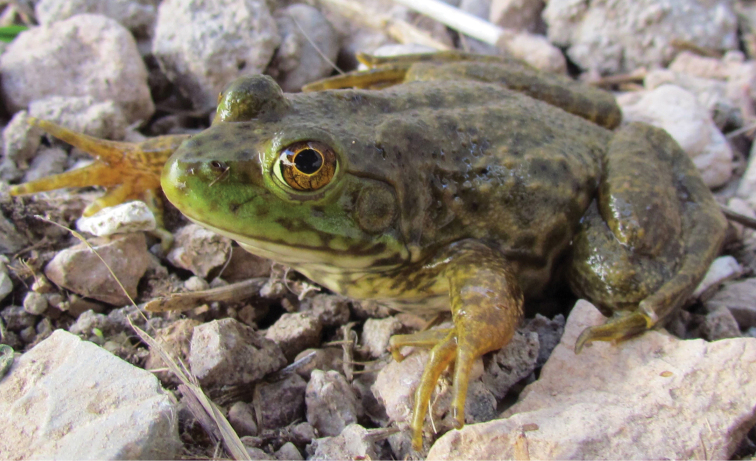
*Lithobates catesbeianus*. Near Chihuahua International Airport, Chihuahua, Mexico. Photograph by Rubén A. Carbajal-Márquez.

Due to a high reproductive rate and generally destructive behaviors, *L. catesbeianus* has great potential for being harmful to native species, and has already been associated with declining and disappearing populations of native amphibians around the world (Casas-Andreu et al. 2001). This frog is considered one of the 100 world’s worst invasive introduced species (Lowe et al. 2000). Also of special concern is the relationship of American Bullfrogs with deadly pathogens, such as *Batrachochytrium dendrobatidis* and *Ranavirus* (Schloegel et al. 2009; Kolby et al. 2014). Recently, an outbreak of *Ranavirus* was reported in captive American Bullfrogs from a farm in Guasave, Sinaloa, in northwestern Mexico (Saucedo et al. 2019). Presently, this pathogen seems not to have spread into wild amphibian populations in Sinaloa, but the risk of *Ranavirus* becoming widespread is high, since there are several susceptible frog species in that area (Saucedo et al. 2019).

***Lithobates forreri* (Boulenger, 1883)**

Forrer’s Leopard Frog’s native distribution was considered to be on the mainland Pacific versant of Mexico from Sonora (Zaldívar-Riverón et al. 2004) into Costa Rica (Savage 2002). Grismer (2002) reported an introduced population in the water systems near Rancho San Juanito within the La Presa region, 100 km north of La Paz, Baja California Sur, suggesting to us a probable intentional translocation from mainland ferry ports across the Sea of Cortes from 1991 to 1993. Those dates were based on personal correspondence between a local rancher and L. L. Grismer (Table 3, Map 25).

### Reptiles – Crocodylia – Crocodiles

#### Family Crocodylidae

***Crocodylus moreletii* (Duméril & Bibron, 1851)**

Fig. 13

Morelet’s Crocodile originally ranged only along the Atlantic lowlands of Middle America, from Tamaulipas, Mexico, to northern Guatemala and adjacent Belize (Cedeño-Vázquez et al. 2012). This crocodile formerly was considered an endangered species and subject to strict conservations measures. Fortunately, in the last few decades, significant recovery of populations has occurred within its native range. There has been an increase in the number of sites dedicated to its conservation by captive breeding, but also for exploiting the animals for food and hides; unfortunately, these farms are mainly found on the Pacific versant of Mexico outside its native range (Álvarez-Romero et al. 2008). As a negative side issue, in Mexico there have been several incidences of *C. moreletii* escaping from these farms into the wild, with populations being established primarily in the states of Oaxaca (Lagunas de Chacahua), Sinaloa, and Colima (Laguna de Alcuzahue) (Álvarez-Romero et al. 2008; Lavín et al. 2014). The first documented case took place during the 1970’s when several Morelet’s Crocodiles were taken from Tabasco to Lagunas de Chacahua, Oaxaca, in order to establish a hide factory there, but after a few years, the project was abandoned and several individuals escaped into the wild (Serrano-Gómez et al. 2016). In addition, the population near Villa Flores in the Central Depression of Chiapas could be an intentional translocation from areas to the north of there (Álvarez-Romero et al. 2008), although that locality is higher up on the Gulf versant with potential riverine access to the lowlands, at least in the past.

**Figure 13.**
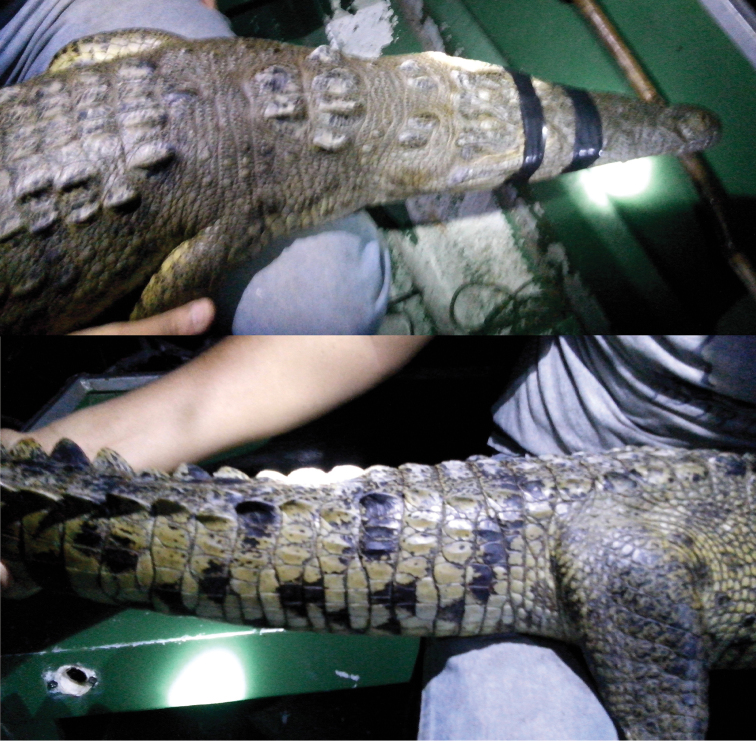
*Crocodylus acutus*. Hybrid pattern (see tail) with *C. moreletii*. APFF Yum Balam, Quintana Roo, Mexico. Photograph by Julio César Gutiérrez-Ramírez.

The invasion of aquatic habitats by *C. moreletii* might have serious consequences for native biotic communities, since it is a large top predator (Álvarez-Romero et al. 2008). Although *C. moreletii* and *C. acutus* (American Crocodile) are sympatric in some areas of their native ranges, in places were *C. moreletii* had been translocated it tended to out-compete and displace *C. acutus* (Lavín et al. 2014). Several cases of hybridization between the two species have been documented from the Mexican Yucatan Peninsula (Cedeño-Vázquez et al. 2008; Rodriguez et al. 2008), Oaxaca (Serrano-Gómez et al. 2016), and Belize (Ray et al. 2004), which is particularly critical for *C. acutus* (Serrano-Gómez et al. 2016), since the genome of *C. moreletii* seems to have a higher fitness value (Lavín et al. 2014). This apparently is true on the Yucatan Peninsula, given the rarity of *C. acutus* in areas where *C. moreletii* is much more abundant. Actions to prevent genomic pollution of American Crocodiles should be encouraged (Cedeño-Vázquez et al. 2008). Furthermore, undocumented crocodiles could become vectors for infectious diseases, such the West Nile Virus (González-Sánchez et al. 2017).

Lazcano-Barrero (1993) acknowledged several intentional releases of *C. moreletii* on Isla Contoy from 1981 to 1991. The individuals came from zoos and from seizures at regional fairs. Very likely, however, those crocodiles emigrated or failed to establish there, since Morelet’s crocodiles are not listed as part of the Contoy herpetofauna (Arriaga y Ramírez-Bautista 2008; González-Sánchez et al. 2017). Also, it is unknown if those released crocodiles contributed to genetic pollution of *C. acutus* populations on Contoy, since studies on hybridization between *C. moreletii* and *C. acutus* on the Mexican Yucatan Peninsula did not include samples from that island (Cedeño-Vázquez et al. 2008; Rodriguez et al. 2008; Machkour-M’Rabet et al. 2009) (Fig. 13).

### Reptiles – Testudines – Turtles

#### Family Emydidae

***Trachemys scripta* (Thunberg in Schoepff, 1792)**

Fig. 14

A common misbelief in Mexico is that the Pond Slider is native to Japan, thus the common name “Tortugas japonesas” (Japanese turtles) is used frequently. The species’ native geographic range, as presently understood, includes the southeastern United States and adjacent lowlands of northeastern Mexico (Rhodin et al. 2017), but due to their intensive husbandry and commercialization as pets, this turtle has become the most widespread chelonian in the world (Standfuss et al. 2016). It is almost impossible to determine exact invasion routes or dates of introduction, since these turtles have been subjected to extensive illegal trafficking, and can easily be purchased in pet stores and markets throughout Mexico (Yáñez-Arenas et al. 2016). Still, it seems highly probable that most introductions in Mexico occurred during the 1980’s through 1990’s, when “Japanese Turtles” gained immense popularity among pet owners.

Any review of literature covering the distribution of this introduced turtle should be made with special care, since the name *Trachemys scripta*, until recently, included almost every Pond Turtle population ranging throughout Middle America, unfortunately, listed as subspecies of *T. scripta* (Campbell 1998; Köhler 2008). Perhaps this is the reason why *T. scripta* appears in the Reptile Database as being introduced into all countries of Central America (Uetz et al. 2020). Johnson et al. (2015b) gave an account of the taxonomic and nomenclatural history of many taxa being considered up until that time, but some of that information has changed. Many subspecies of Pond Turtles recently have been rightly elevated to full species, thereby making them native populations to their inclusive ranges throughout Middle America (e.g., Seidel 2002; Fritz et al. 2012; McCranie et al. 2013; Parham et al. 2013, 2015). According to Parham et al. (2015), the native species in Middle America that occurs geographically closest to *T. scripta* is *T. venusta* in northeastern Mexico, which in itself has gone through taxonomic reorganization, so today its range is primarily restricted to the Atlantic versant of Middle America into northern South America.

Our review of introduced species will only cover what has been called the Red-eared Slider, *T. scripta elegans* (Wied, 1838), which is listed among the 100 most dangerous invasive species, according to the Invasive Species Specialist Group (Lowe et al. 2000), although we herein do not consider subspecies as a legitimate formal taxonomic category (see Johnson et al. 2015b), but only “pattern classes” of geographic variants exhibited within a single species (Grismer 2002). The Elegans pattern class individuals of *T. scripta* are of special concern due to their deleterious tendencies to outcompete other turtles for basking sites, and as possible vectors for spreading diseases (Lavín et al. 2014).

Established introduced populations of the Elegans pattern class of *T. scripta* are disconnectedly distributed in several parts of Mexico, such as in Baja California, within several northern states, along the southern Pacific slopes, and on the Yucatan Peninsula (Lavín et al. 2014). We must clarify that the latest revision of the herpetofauna of the Mexican Yucatan Peninsula (González-Sánchez et al. 2017) failed to mention *T. scripta*, since it was thought that distributional records at that time from the peninsula corresponded to its close relative, *T. venusta*. These authors did overlook the report published by Böehm (2013), however, for some turtles from populations living within cenotes on the Yucatan Peninsula that clearly resembled the Elegans pattern class of *T. scripta*.

In Honduras, *T. scripta* has been observed in Río Llanitos, Santa Bárbara, and Isla Guanaja in the Islas de la Bahía (= Bay Islands) (McCranie et al. 2005; Solís et al. 2014). McCranie and Valdés-Orellana (2014), however, did not know whether the few known specimens from Guanaja were part of an established population, or merely individual escaped pets. McCranie and Valdés-Orellana (2014) also specifically mentioned a female from Isla de Guanaja at Savannah Bight (FMNH 283584) that was suspected to be an escaped pet because both *T. scripta* and *T. ornata* (= *T. venusta*; Parham et al. 2013, 2015) are regularly kept as pets by local citizens. Another report was published by McCranie and Valdés-Orellana (2014) of a vouchered, but supposedly uncatalogued *T. scripta* in the collection of UNAH from 11.9 km north of Cofradía, Cortés, on the mainland, located 290 km southwest of Savanna Bight on Isla Guanaja.

**Figure 14.**
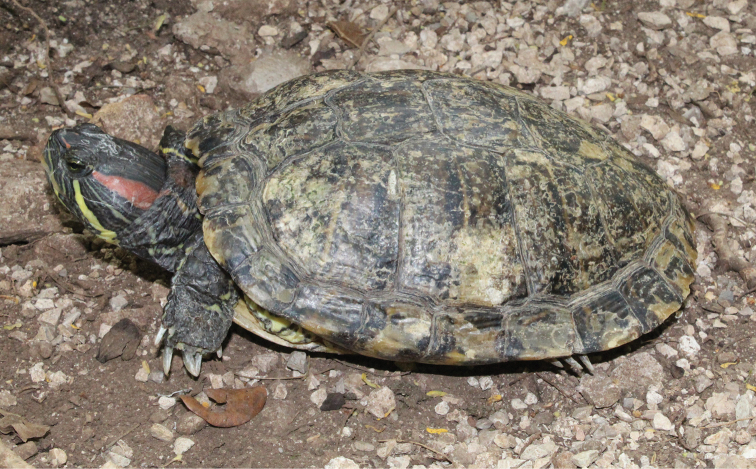
*Trachemys scripta elegans* (Elegans pattern class of *T. scripta*). Xcunya, Yucatan, Mexico. Photograph by Luis Díaz-Gamboa.

Kraus (2009) listed the red-eared slider (Elegans pattern class of *T. scripta*) as introduced in Panama, citing Moll (1995), but that reference was an editorial letter in which the author only mentioned having collected specimens in Panama; thus, he didn’t provide any specific locality, date, or voucher specimen. Jaramillo et al. (2010) did not list *T. scripta* as an introduced species in Panama. Therefore, we have found no verified report for any established populations in Panama (Figure 14).

***Trachemys venusta* (Gray, 1855)**

The Mesoamerican Slider, as discussed elsewhere, was involved in the taxonomic dispute associated with *T. ornata* (Fritz et al. 2012; Parham et al. 2015). Presently, *T. ornata* and *T. venusta* are considered as separate species, with *T. ornata* ranging along the Pacific lowlands of Mexico from Sinaloa to at least southeastern Guerrero, and *T. venusta* ranging from Tamaulipas, Mexico, on the Atlantic lowlands into South America (Parham et al. 2015; Legler and Vogt 2013). Much of the taxonomic controversy involved slider turtles sampled from the vicinity of Acapulco, Guerrero, which Parham et al. (2015) showed to be translocated *T. venusta*. We agree with their conclusion.

#### Family Kinosternidae

***Kinosternon integrum*** (Le Conte, 1854)

A single individual of the Mexican Mud Turtle was captured 29 March 2007 in a perennial pool at the bottom of a rocky canyon in the Sierra La Laguna, Baja California Sur (Luja et al. 2007). Apparently, this was one of a pair mentioned by a local settler as being released into the pool by someone else, most likely in the second half of the 1980’s; the other turtle was found dead at the site approximately ten years after its original release. Luja et al. (2007) made no mention about where the turtles originated. We assume the turtles were translocated by someone from the mainland on the Pacific versant of northwestern Mexico. Ferryboats regularly carry people across the Sea of Cortes from the Port of Pichilinque, near La Paz, Baja California Sur, to ports in Sinaloa at Topolobampo (near Los Mochis) in the northwest and Mazatlán in southwestern portion of the state. For now, we assume an established translocated population is probable at this site.

Iverson et al. (1998) stated that *K. integrum* populations in the Valley of Mexico were introduced, but they did not provide arguments to support that idea. It was also suggested that the probable extinction of *K. hirtipes hirtipes*, a supposed endemic subspecies of mud turtle known only from three to five localities in the Valley of Mexico (Legler and Vogt 2013; Rhodin et al. 2017), “may or may not be associated with the introduction of *K. integrum*, which has become very common there” (van Dijk et al. 2007); no evidence or explanation was given to confirm that assessment. Ramírez-Bautista et al. (2009) indicated that *K. integrum* and *K. hirtipes* are sympatric in Lago de Xochimilco (Distrito Federal), but they did not indicate if *K. integrum* was introduced or that *K. hirtipes* had become extinct there. *Kinosternon integrum* also inhabits Canal de Chalco, in the state of Mexico, and *K. hirtipes* also occurs in Lago de Tlahuac. We assume for now that both species have established populations in the Valley of Mexico, and that *K. integrum* was introduced there.

#### Family Trionychidae

***Apalone spinifera* (Le Sueur, 1827)**

The Spiny Softshell has a limited native range in Mexico, principally in drainage systems associated with the Rio Grande, which is the border with Texas and a very small segment of New Mexico near El Paso, with neighboring states in Mexico (Chihuahua, Coahuila, Nuevo León, and Tamaulipas), then continuing along the Gulf lowlands to approximately Soto la Marina, Tamaulipas (Legler and Vogt 2013). Farr (2015) apparently considered this turtle native throughout Tamaulipas, as did Lemos-Espinal and Dixon (2013) for the record of *A. spinifera* in San Luis Potosí. This turtle has been reported four times from translocated populations in northwestern Mexico (Rorabaugh and Lemos-Espinal 2016), once in the lower basin of Río Colorado and its drainages in the Mexicali Valley, Baja California (Grismer 2002), and three times in Sonora, twice from the Welton Canal area in the southeastern Río Colorado Valley (Rorabaugh et al. 2008) and once from the Río San Rafael in the Municipality of Cananea, 449 km to the east-southeast (Rorabaugh and King 2013). The introductions in Sonora are probably the result of expansion of an invasion front that might have originated by intentional introductions of Spiny Softshell turtles (together with fishes and frogs) by ranchers along the Gila River early in the 20^th^ century. From there, the range expanded until reaching the lower Colorado Basin and Mexicali Valley (Miller 1946), where reportedly they were once plentiful, but now in decline due to hunting pressures (Mellink and Ferreira-Bartrina 2000); Spiny Softshell meat has been served frequently in Chinese restaurants in Mexicali (Grismer 2002) (Table 3, Map 26).

In the Cuatro Ciénegas area of Coahuila, Mexico, *A. spinifera* arrived at some local water sources when irrigation channels were opened from the northeast in the 1880’s (McGaugh and Janzen 2008). It has been linked to the impending extinction of the endemic Black Softshell (*A. atra*) due to hybridization, although this claim is yet to be fully confirmed, since “pure” individuals of *A. atra* could still be found during the 1970’s and 1980’s (Webb 1973; Legler and Vogt 2013), especially at the type locality. McGaugh and Janzen (2008) thought that there was insufficient molecular evidence to differentiate the two turtles and concluded they were conspecific, but Bonin et al. (2006) and Legler and Vogt (2013) regarded them as separate species. Wilson and Johnson (2010) reviewed the evidence and decided to continue recognizing *A. atra* until a study showing complete genetic introgression between all populations of the two species refutes the claim that they are separate evolutionary lineages. We agree with that conclusion because of historical genetic isolation and the reasons enunciated by Wilson and Johnson (2010). If it turns out to be true that a translocated invasive population of *A. spinifera* has hybridized to the point of full genetic introgression with *A. atra*, then, unfortunately, this situation will become a prime example of genetic extinction of a formerly endemic species by hybridization with a non-endemic species.

Outside of northern Mexico, a single record exists for *A. spinifera* from Jalisco in an artificial pond in Puerto Vallarta (Cupul-Magaña 2012), but nothing was said about it being from an established population. According to F. G. Cupul-Magaña (pers. comm.), the turtle was still alive as of August 2018 and living in an open-air aquarium in Puerto Vallarta, but he does not know the original date of translocation or where it originated. Cruz-Sáenz et al. (2017) did not list *A. spinifera* as a member of the Jalisco herpetofauna, presumably because they did not consider it an established population; we agree with that conclusion.

There are, however, documented localities in Guerrero for *A. spinifera* from along the Río Balsas drainage, one from the vicinity of Colonia Valerio Trujano, near the Mezcala Bridge, Municipality of Edwardo Neri, that seems to be from an established population. Local residents indicated that it had been present there since the 1950’s (Lemos-Espinal et al. 1999). Two turtles were taken from that area and according to the authors, were deposited in the Herpetology Collection, Unidad de Biología, Tecnología y Prototipos, UNAM, Campus Iztacala, Tlalnepantla, Estado de México. The other locality that allegedly has an established population is from the Municipality of Copalillo, 5 km northeast of Papalutla at the edge of Río Atoyac; a specimen from there was deposited in the Colección del Laboratorio de Herpetología Vivario, Facultad de Estudios Superiores Iztacala, UNAM (CLHV 4462-E) (Jiménez-Arcos et al. 2009).

Castro-Franco and Zagal (2004) reported an adult female *A. spinifera* captured 17 October 1999 in the Río Amacuzac on the Sierra de Huautla Natural Protected Area near Las Huertas Spa, Municipality of Tlaquiltenango, Morelos, which was deposited in the Herpetological Collection of the Universidad Autónoma del Estado de Morelos (EBUM 2898). Another turtle was observed previously in that same Protected Area in Cruz Pintado Pond, but was not captured. Those turtles indicate a probable established population, and were thought to have been released pets.

García-Vázquez et al. (2009) first reported *A. spinifera* as occurring in Puebla, Mexico, without commenting about it being introduced to the state or not. Most recently, Woolrich et al. (2017) considered the Puebla record as an introduction, a determination with which we agree.

### Reptiles – Squamata – Lizards

#### Family Iguanidae

***Ctenosaura conspicuosa* (Dickerson, 1919)**

The Isla San Esteban Spiny-tailed Iguana only exists on Cholludo and San Esteban islands, in the Sea of Cortes, located in close proximity to the coast of Sonora, Mexico. The cultural evidence suggests that *C. conspicuosa* populations on both islands could be due to a prehistoric introduction of *C. nolascensis* from Isla San Pedro Nolasco by the Seri culture (Grismer 2002; Nabhan 2002). The divergence time between those two lineages, however, is much older (~890, 000 years), which coincides with the detachment of Isla San Esteban from mainland Sonora (Edwards et al. 2005). Even so, there seems to be enough molecular and cultural evidence to indicate that the population of *C. conspicuosa* present on Isla Cholludo was introduced indeed from Isla San Esteban by the Seri culture (Buckley et al. 2016) (Table 3, Map 27).

***Ctenosaura pectinata* (Wiegmann, 1834)**

The Western Spiny-tailed Iguana naturally occurs in low to intermediate elevations primarily on the Pacific versant of Mexico from Sinaloa into Chiapas, including subhumid interior basins and valleys and offshore islands (Uetz et al. 2020). This iguana was introduced on the remote Isla Clarion sometime in the mid-1990´s (Aguirre-Léon and Matías-Ferrer 2017; CONANP 2018), although the path of introduction is unknown. We can be sure that they were introduced, since spiny-tailed iguanas are conspicuous animals, and not shy around human presence; thus, they should be noted easily, but were not reported in previous herpetofaunistic listings for that island (Townsend 1890; Brattstrom 1955, 1990). Whether there is an impact of *C. pectinata* on native species, such as the endemic *Urosaurus clarionensis* is unknown (Table 3, Map 28).

***Ctenosaura similis* (Gray, 1831)**

Fig. 15

The Black Iguana naturally occurs on the Atlantic and Pacific versants from Mexico, below the Isthmus of Tehuantepec, through all countries in Central America (McCranie et al. 2005; Köhler 2008; Buckley et al. 2016). This lizard was reported as introduced to Isla Roatán, Honduras, where it was detected in 2012 (Pasachnik 2013); apparently, the site of initial translocation was on a small island off the south coast of Roatán near Coxen Hole. The Black Iguana could be an immediate threat to native *C. oedirhina*, since it might compete for resources and/or hybridize (McCranie and Valdés-Orellana 2014). Probably other insular populations of *C. similis* in the Caribbean waters of Mexico and Central America are intentional introductions, as exemplified by Sunyer et al. (2013), who mentioned probable translocations of *C. similis* and *Iguana iguana* as food sources for residents of the Corn Islands, Nicaragua, although this opinion was not based on empirical fact. A Mexican Navy cadet told one of us (VHGS) that both the Black Iguana and Green Iguana were introduced several decades ago as ornamental species by navy cadets in Banco Chinchorro, Quintana Roo, Mexico, but there is no way to verify that claim. Thus, except for the Black Iguana population on Roatán, we do not consider the other locations as having confirmed translocations (Table 3, Map 29).

**Figure 15.**
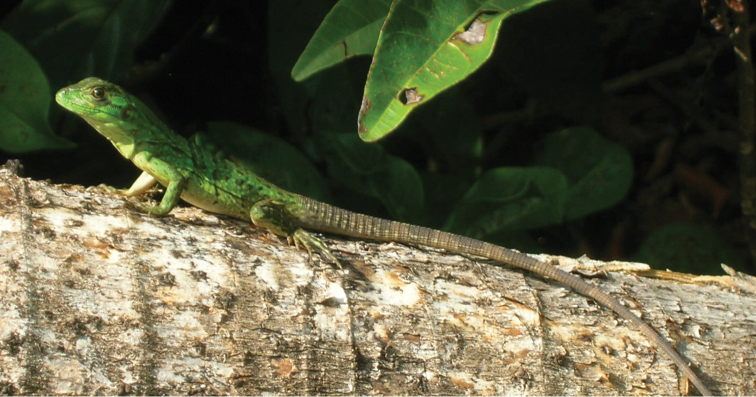
*Ctenosaura similis*. A juvenile specimen from Little Corn Island, Nicaragua. Photograph by Javier Sunyer.

***Sauromalus hispidus* (Stejneger, 1891)**

The Spiny Chuckwalla inhabits several islands within the Gulf of California, including: Angel de La Guarda, Alcatraz, Cabeza de Caballo, Flecha, Granito, Mejía, Piojo, Pond, San Lorenzo Norte, San Lorenzo Sur, and numerous islands in Bahía de Los Ángeles (Grismer 2002; Buckley et al. 2016); a single individual also was reported from Isla Rasa (Velarde et al. 2008). The Spiny Chuckwalla was probably introduced from Ángel de la Guarda onto La Ventana and Smith islands, since individuals from both populations have “a nearly identical cytochrome b sequence as some Ángel de la Guarda *S. hispidus*.” The species also could have been translocated to San Lorenzo Sur, but the possibility exists that there was a previous land bridge involved (Petren and Case 1997). The Spiny Chuckwalla population on Isla Alcatraz might encompass introduced hybrids (Grismer 2002), since hybridization and genetic introgression have been suggested among *S. hispidus*, *S. varius*, and *S. obesus* (Robinson 1972). Kraus (2009) indicated a date of introduction to be around 1920, but he didn’t give concrete reasons for that estimation, and overlooked the much earlier cultural evidence of insular translocations by Seri navigators (Nabhan 2000, 2002). Shaw (1946) pointed out that some introductions probably occurred naturally due to Ospreys (*Pandion haliaetus*) inadvertently dropping live chuckwallas into their nests; he also was aware of translocations of individuals by the Seri culture as food sources (Table 3, Map 30).

***Sauromalus varius* (Dickerson, 1919)**

The Piebald Chuckwalla is only known from the islands of San Esteban and Roca Lobos in the Sea of Cortes (Grismer 2002). Hollingsworth et al. (1997) reported the first record for Roca Lobos, which they considered an introduced population based on the lack of morphological divergence between that population and the one on San Esteban. They also thought that natural colonization seemed unlikely since Isla Salsipuedes, with no *S. varius*, lies between the two islands and would have represented a barrier to direct overwater dispersal. Hollingsworth et al. (1997) also pointed out that the *S. varius* population on Roca Lobos could have been an intentional introduction by researchers attempting an experiment on the effects of colonization and evolution of insular populations. Considering the decades of studies they quoted, the introduction might have occurred in the midpoint or end of the 1970’s. A decade after the initial report, the Isla San Esteban Chuckwalla population was reported as being “healthy and reproducing” (Lovich and Mahrdt 2007) (Table 3, Map 31).

#### Family Phrynosomatidae

***Uta stansburiana* (Baird & Girard, 1852)**

The Side-blotched Lizard is a common widespread generalist, occurring in the western United States, northern Mexico, and along the Baja California Peninsula and many of its associated islands (Grismer 2002). Interestingly, *U. stansburiana* is present on islands in the Sea of Cortes and Pacific Ocean, and on those with both continental and oceanic origins (Murphy and Aguirre-León 2002), some of which also are associated with endemic species of *Uta* (Grismer 2002). Certainly, many of those populations are natural overwater dispersal colonizers and, taking into account the anthropological evidence of translocations, some insular populations, assuredly, could be introduced (Murphy and Aguirre-León 2002). This assertion seems to be supported at least by the *Uta* populations on Isla La Raza, a tiny volcanic island in the Sea of Cortes, where molecular evidence suggests colonization from the north, from Ángel de la Guarda and/or Isla Mejía populations, something that Murphy and Aguirre-León (2002) considered unlikely by natural over water current dispersal, since the severe upwellings in that area should impede that movement. Additionally, the anthropological evidence of human occupancy on Isla La Raza and the lack of genetic differentiation points to accidental introduction, probably during prehistoric times (Upton and Murphy 1997) (Table 3, Map 32).

### Reptiles – Squamata – Snakes

#### Family Boidae

***Boa imperator* (Daudin, 1803)**

The Central American Boa Constrictor, formerly a subspecies of *Boa constrictor* (Reynolds et al. 2014), is widespread in Middle America, ranging on the Atlantic versant of Mexico to the Isthmus of Tehuantepec and on both Atlantic and Pacific sides below the Isthmus to northwestern Colombia (Card et al. 2016; Suárez-Atilano et al. 2017). Despite being native on the mainland Caribbean versant of Mexico, including the Yucatan Peninsula and several offshore islands, it was considered an introduced invasive species on Cozumel Island, Quintana Roo (Martínez-Morales and Cuarón 1999; González-Sánchez et al. 2017), although reasons for its distributional status were controversial as late as 2008 (Álvarez-Romero et al. 2008). It is clear now that *B. imperator* was unknown on Cozumel until 1971, when according to local independent informants, cinematographers filming the movie “El Jardín de la Tía Isabel” released several boas of various sizes in order to create a more “exotic” atmosphere (González-Sánchez et al. 2017). This conclusion was reinforced by the fact boas had not appeared in any biological listing until the inventory made by López-González (1991). A molecular study by Vázquez-Domínguez et al. (2012) documented the existence of a founder effect in the genome of Cozumel’s *B. imperator* and close phylogenetic ties with populations on the adjacent mainland.

Martínez-Morales and Cuarón (1999) suggested a possible link between the arrival of *B. imperator* on Cozumel with the decline of several native species, such as the Cozumel Thrasher (*Toxostoma guttatum*), Central American Agouti (*Dasyprocta punctata*), Cozumel Raccoon (*Procyon pygmaeus*), Cozumel Coati (*Nasua narica*), and the Cozumel Curassow (*Crax rubra*), among others. Of those, the Cozumel Raccoon is under a highly critical extinction risk (de Villa-Meza et al. 2011). Thus, it is imperative that a plan should begin now to eliminate or tightly manage this invasive snake species that is threatening Cozumel’s wildlife. We are unaware of any attempted *B. imperator* control program on Cozumel Island, however, beyond surveys or ecological studies.

Charruau et al. (2015) suggested that *B. imperator* might be alien to Cayo Centro (Banco Chinchorro, Quintana Roo), since it is a large reptile and not seen previously by local anglers, although the management program for that reserve already listed this snake as occurring there. A molecular analysis, however, should be done in order to clarify the origin of the Cayo Centro population (Table 3, Map 33).

### Reported introduced species not on our list of established populations in Middle America

***Taricha torosa* (Rathke, 1833)**

Murphy and Méndez de la Cruz (2010) listed the California Newt (Salamandridae) as introduced into Baja California, but they did not provide any details or explanations. Grismer (2002) considered it a species that probably occurs in northern Baja California. Kuchta (2005) listed it based on supposed records for northwestern Baja California by Slevin (1928), Smith and Taylor (1948), and others, but all of those records need verification. *Taricha torosa* definitely occurs naturally in San Diego County, California, a few kilometers from the Mexican border (Kuchta 2005), therefore, the most probable scenario of potential extant Mexican populations reflects natural range expansions from the San Diego populations. Thus, at this time, we do not consider it a native or an introduced species in Mexico.

***Gopherus agassizii* (Cooper, 1861)**

The taxonomic history of the *G. agassizii* species complex of Desert Tortoises (Testudinidae) generally had been unresolved (Murphy et al. 2011). Until recently, only *G. agassizii* was officially recognized and naturally resided in the Mojave and Sonoran Deserts in the USA and Mexico, southward in Sonora (including Tiburón Island) into areas of Sonoran desertscrub, Sinaloan thornscrub, and tropical deciduous forest to around Alamos, Sinaloa. Reported populations in Baja California and Baja California Sur were mostly associated with introductions (Bury et al. 2002; Murphy et al. 2011; Legler and Vogt 2013). The species complex has undergone recent taxonomic revisions (Murphy et al. 2011; Edwards et al. 2016) and future studies might well lead to additional taxonomic modifications.

Murphy et al. (2011) removed *G. agassizii* (Agassiz’s Desert Tortoise) from the herpetofauna of Mexico by formally describing *G. morafkai* (Morafka’s Desert Tortoise). *Gopherus morafkai* also ranges into the USA, primarily in Sonoran Desert areas in Arizona south and east of the Colorado River. *Gopherus agassizii* is now considered native to the Mojave Desert of California, Nevada, Utah, small areas in northwestern and southwestern Arizona, and a small section of the Sonoran Desert in southeastern California. Edwards et al. (2016) further divided the Mexican populations into *G. morafkai* and a new species, *G. evgoodei* (Goode’s Desert Tortoise), which is endemic to Mexico and native to primarily Sinaloan thornscrub and tropical deciduous forests in east-central and southern Sonora and northern Sinaloa. The two species occasionally hybridize in the ecotone between Sonoran desertscrub (*G. morafkai* preferred habitat) and Sinaloan thornscrub (*G. evgoodei* preferred habitat); no hybrids were observed from tropical deciduous forest habitats.

Ottley and Velazquez-Solís (1989) described a new species of tortoise (as *Xerobates lepidocephalus*), based on one live individual and shell remains of another, from the Cape region of Baja California Sur, specifically from the Sierra San Vicente, 1.5 km north of the Buena Mujer Dam, 20 km south of La Paz. Crumly and Grismer (1994) noted that its morphological variation fell within that expected for individuals from Sonoran populations, thus they considered *X. lepidocephalus* as a junior synonym of *G. agassizii*; they further opined that the population was a probable introduction. Grismer (2002), however, considered the possibility of a native relict population existing in the Cape region that later was reduced considerably when goat grazing was allowed. To us, it also seems plausible that the construction of Buena Mujer Dam in the 1980’s would have destroyed a large area of suitable tortoise habitat, leaving only a few survivors. Murphy et al. (2011) inconclusively pointed out that the holotype of *X. lepidocephalus* might correspond to a hybrid, so if this is the case, it supports the hypothesis of an introduced population, since hybrid lineages are common in non-native species (Edwards et al. 2010). The hybrids most likely would be between *G. morafkai* and *G. evgoodei* that originated as pets taken to the La Paz area. On the other hand, if a population of native tortoises exists in the La Paz region, then they certainly should be considered a separate evolutionary species (Murphy et al. 2011), because of its allopatric distribution compared to that of *G. agassizii*, *G. morafkai*, and *G. evgoodei*. In that case, measures to guarantee the population’s protection should be made available as soon as possible (Murphy and Mendez de la Cruz 2010).

At this point, we do not think that the population around La Paz has been identified definitively as being *G. morafkai*, *G. evgoodei*, *G. agassizii*, or one of hybrid origin. We also consider any inhabitants that will be found in northeastern Baja California will probably fall within the native range of *G. morafkai*, unless they can be shown positively to be part of translocated *G. agassizii*, *G. evgoodei*, or hybrid populations. Thus, it will not be appropriate at this time to list any population of the *G. agassizii* species group of Desert Tortoises as being introductions within northwestern Mexico.

***Gopherus berlandieri* (Agassiz, 1857)**

A single specimen of the Texas Tortoise (Testudinidae) was found in a city park at Puerto Vallarta, Jalisco, Mexico (Cupul-Magaña 2012). Cruz-Sáenz et al. (2017) did not consider it as part of the Jalisco herpetofauna, nor is there any other record for this species outside its native range in northeastern Mexico, so we concur that it was not part of an established population.

***Staurotypus triporcatus* (Wiegmann, 1828)**

Until recently, the Mexican Giant Musk Turtle (Staurotypidae) was considered native to the Atlantic lowlands from central Veracruz, Mexico, through the southern Yucatan Peninsula, and on to the western Caribbean lowlands of Honduras (Legler and Vogt 2013; Rhodin et al. 2017). Terán-Juárez et al. (2015) reported this turtle near Ocampo, Tamaulipas, Mexico, ca. 524 km to the north of the closest known locality in central Veracruz. They considered this record the result of an introduction due to the large hiatus between those localities. Terán-Juárez et al. (2016), however, later regarded the population as native because there was no empirical evidence to support its translocation by human activities from farther down the Gulf Coastal Plain. Our experience with *S. triporcatus* indicates that individuals rarely leave water sources and cross roads like other kinosternid turtles, especially those in the genus *Kinosternon* and even *Claudius*. Legler and Vogt (2013) also mentioned that *S. triporcatus* in Belize never were observed on land during its activity season. Crossing roads could be a good source for translocating turtles along roadways, but if that rarely happens with *S. triporcatus*, the capability of being translocated is diminished. Until additional information indicates otherwise, we agree with Terán-Juárez et al. (2015) that the records came from a marginal area of its native range in northeastern Mexico.

***Trachemys ornata* (Gray, 1831)**

The *Trachemys scripta* species group (Emydidae) has had a confusing taxonomic history in Middle America, especially those populations occurring in tropical latitudes (Johnson et al. 2015a), so more work is required to properly determine native species boundaries (Parham et al. 2013, 2015). The Ornate Slider is presently considered a Mexican endemic ranging on the Pacific lowlands of Mexico below 300 m elevation from Sinaloa to 4 km northwest of Ixtapa, Guerrero, which is ca. 220 km northwest of the Acapulco, Guerrero area (Mertz et al. 2015). This turtle also was reported to occur in several lagoons around Acapulco and sold for food in local markets (Legler and Vogt 2013). According to Parham et al. (2015), however, all samples they evaluated near Acapulco were *T. venusta*, which they considered translocations from the Atlantic lowlands of Mexico. Rhodin et al. (2017) alleged that an isolated record of *T. scripta* (= *T. ornata*?) in Michoacán and other remote records on the Pacific lowlands in Guerrero might have been introduced as well. A connection between the coastal lagoon systems on the Pacific lowlands, however, might have existed in the past (Legler and Vogt 2013), and possibly still does, so it seems likely to us that the range of *T. ornata* might extend even farther down the Pacific lowlands past the Acapulco region. We will not include *T. ornata* as anything other than native until the origins and taxonomic status of those Pacific lowland populations are fully resolved.

***Cnemidophorus ruatanus* (Barbour, 1928)**

The Ruatan Whiptail (Teiidae) was reported (as *Cnemidophorus lemniscatus*) by Stafford and Meyer (2000) from Monkey River town, Toledo District, Belize; Stafford et al. (2010), without comment, considered it as an introduced species. McCranie and Hedges (2013) elevated *C. ruatanus* to species level from its previous status as a subspecies of *C. lemniscatus*. Due to the proximity of this record to other known localities, however, and for maintaining consistency in a coastal scheme of distribution (McCranie and Hedges 2013), we consider this population as being a marginal natural expansion of its total distributional range.

***Gonatodes albogularis* (Duméril & Bibron, 1836)**

Fig. 16

Identifying the native distribution of the Yellow-headed Gecko (Sphaerodactylidae) is problematical because of its wide range in parts of Middle America, northern South America, and on many islands in the West Indies (Uetz et al. 2020). A pertinent question is whether its native range is restricted to Middle and South America and it is introduced in the West Indies, or vice versa. Stuart (1963) was under the impression that all West Indian records were introductions. Villa et al. (1988) and Köhler (2008) did not mention it ranging into the West Indies, which we infer to mean that those individuals were not considered native to that area. Others have apparently included the West Indies as part of the natural range (e.g., Lee 2000; Savage 2002; McCranie et al. 2006; Johnson et al. 2010); most of those sources also reported *G. albogularis* as being introduced into Florida, without referring to its possible origin.

**Figure 16.**
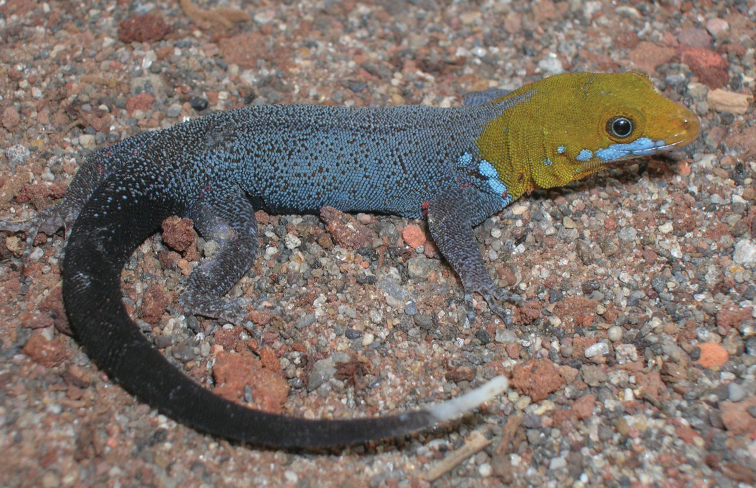
*Gonatodes albodularis*. Ometepe island. Nicaragua. Photograph by Javier Sunyer.

On the mainland, *G. albogularis* occurs from the Pacific slopes of the Soconusco and Sierra Madre regions in Chiapas, Mexico, through Central America into northern Colombia and Venezuela (Johnson et al. 2015a), at elevations lower than 1,000 m. Its northernmost locality on the Atlantic versant of Mexico recently was reported as Minatitlán, Veracruz, by Guzmán-Guzmán and Palma-Martínez (2016). It was discovered in Belize City in 1996 (Lee 2000) and Stafford et al. (2010) thought it had been introduced recently into that country, but with no substantiated evidence. Until new information demonstrates otherwise, we regard *G. albogularis* as being native to Middle and South America and exotic in the West Indies and Florida.

***Phyllodactylus nocticolus* (Dixon, 1964)**

The occurrence of Peninsular Leaf-toed Gecko (Phyllodactylidae) on Isla Tiburón could be due to natural over-water island hopping, accidental introduction (Murphy and Aguirre-León 2002), ancient historical translocation from other islands in the Sea of Cortes (Nabhan 2000), or even by paleotectonic activity (Blair et al. 2009). Without specific evidence to support any of the above-mentioned scenarios, we elect not to list this species as introduced at this time, but it is certainly a candidate worth further investigation.

***Phyllodactylus xanti* (Cope, 1863)**

Mellink (2002), citing Nabhan (2002), indicated that the Cabo Peninsular Leaf-toed Gecko (Phyllodactylidae) was introduced involuntarily from mainland Baja California onto San Lorenzo, San Esteban, Tiburón, and Alcatraz islands, probably by boats. We could not find any corroborating reference or study suggesting that scenario. Thus, because we could not specifically determine if the *P. xanti* population on Tiburón Island originated there by natural dispersal or by some sort of human-mediated introduction process (see *P. nocticolus* account above), we decided not to include this species on our list of introductions at this time.

***Sauromalus obesus* (Baird, 1859)**

A chance exists that the Western Chuckwalla (Iguanidae) might have been translocated to several islands in the Sea of Cortes, based on the same reasons as those for *S. hispidus* and *S. varius* (Nabhan 2002), but not enough evidence was presented to support that contention. Therefore, we are awaiting more verification before deciding to place it on our list of species introduced into Mexico.

***Varanus exanthematicus* (Bosc, 1792)**

The Savanna Monitor (Varanidae) is known in Mexico from a single record in Puerto Vallarta, Jalisco (Cupul-Magaña 2012). This record probably corresponds to an escaped pet or intentional release. We know of no other record from anywhere else in Middle America, so we do not consider the lizard as coming from an established population.

## Considerations for management and control measures for introduced species

The main difficulty for management and control of the introduced invasive herpetofauna of Middle America is the lack of documentation. Most conservation managers or agencies have little to no tradition of publishing in the refereed scientific literature, so, for the most part, successes and failures of invasive species are found in grey literature sources (Simberloff 2009). Even more daunting is the fact that among known eradication/management programs cited in the literature, few concern reptiles (Campbell III et al. 1999; Toda et al. 2010) or amphibians (Miller 2006; Greenlees et al. 2018), and hardly any evaluate effectiveness of their control techniques (Rodda et al. 1999; Davis et al. 2015; Haramura et al. 2017; Muller and Schwarzkopf 2017). The main consequence of this “nothing can be done” approach is the lack of evidence for successful programs (Simberloff 2009). Therefore, the first challenge must be to encourage managers to publish their results in the scientific literature.

A key component of any successful control or eradication program is early detection of an invasion and a quick response (Simberloff 2009). Unfortunately, only in limited cases are introductions detected early, so usually dates are merely approximations or suppositions. The true origins of many species are often uncertain, especially if they are common, and times and places of the introduction are unknown; many are commonly considered native even though their true place of origin is unresolved. Those instances refer to the “cryptogenic species” of Carlton (1996), a good example of which is the status of *A. allisoni* being listed as native to the Yucatan Peninsula of Mexico (González-Sánchez et al. 2017), Belize (Stafford et al. 2010), and Honduras (Solís et al. 2014; McCranie 2015), even though others consider them introductions, including Glor et al. (2005) and Álvarez-Romero et al. (2008). Additionally, in some situations, some apparent “endemic” species might be, in fact, the result of an ancient anthropogenic introduction of an alien species; this situation can occur particularly in insular ecosystems after thousands of years of isolation, and are referred to as “ethnospecies” (Hofman and Rick 2018). This explanation could apply to some endemic iguanids (such as *Sauromalus* and *Ctenosaura*) on islands in the Sea of Cortes; there has been speculation about the origin of the population of *C. conspicuosa* on San Esteban island, which is believed to have originated from translocated individuals of *C. nolascensis* from San Pedro Nolasco Islands by the Seri people (Grismer 2002). Davy et al. (2011), however, refuted this notion and specified that *C. nolascensis* and *C. conspicuosa* diverged thousands of years before human colonization of the Sea of Cortes. Still, the idea should not be discarded, as some iguanid endemic lineages on those islands might be ethnospecies.

Another key component of control and eradication programs involves the correct identification of the presumed introduced species by qualified specialists and their training of non-expert volunteers so to avoid misidentification of native species as introduced ones. These specialists also need to participate in the removal programs. These lessons have come from the work of Rick Shine and his colleagues on the Cane Toad or Marine Toad (*Rhinella marina*) in Australia, as reported in numerous publications, including the book *Cane Toad Wars* (Shine 2018). Nonetheless, the work of Shine and his colleagues has demonstrated that simple, physical removal of adult toads is ineffective in reducing their numbers or in curtailing their spread through Australia and that what is more promising is an approach using integrated pest control (Shine 2015). Shine (2015: 312) indicated that “Cane toads are formidable invasion machines, and it is unlikely that any single method will ever eradicate them. Even with a combination of methods, landscape-scale extirpation is vanishingly unlikely. However, the new weapons developed out of recent ecological research on this high-profile invasive anuran provide great encouragement.” Shine (2015: 314) further noted that the release of “juvenile (and thus, non-lethal) toads at the current invasion front [can be employed] to train native predators to avoid toads as prey” and that “funnel-traps baited with toad toxins can eradicate toad tadpoles from natural water bodies.” In general, he concluded that “cane toads in Australia provide a clear example that [one needs] to understand an invasive species if [one wants] to control it.” This “whole-biology” approach also is stressed by Tyler (2006) in his discussion of the potential use of pheromones of both tadpoles and adults in the control of invasive populations of this toad.

A common challenging situation emerges in cases where some translocated species are under legal protection, but are exotic or even invasive in other parts of the country. This situation keeps conservation managers from performing effective eradication or control measures on those invasive populations (Lazcano et al. 2010). For example, Morelet’s Crocodile and *Boa imperator* (as *Boa constrictor*) appear under the categories of “subject to special protection” and “threatened,” respectively, by Mexican legislation (D.O.F. 2015). Obviously, when describing an invasive species, its location of controlling action should be strictly identified (Richardson et al. 2011), or an accurate delineation of both the native areas where the species resides and where it is invasive be given (Ochoa-Ochoa et al. 2017).

The first obvious method for controlling introduced species of amphibians and reptiles is direct capture/sacrifice and trapping methodology. Some biologists, however, are opposed to the sacrificing such introduced creatures, for ethical reasons. This position is entirely understandable and is complex enough to require adequate discussion elsewhere. Several capture techniques include using nooses, pitfall traps, funnel traps, sticky traps, rubber bands, firearms, blowguns, and road cruising, among others, whose effectiveness are well known to herpetologists, thus we will not detail them here. Less common, but potentially successful procedures are discussed below.

Chemical control has a long tradition for managing invasive mammals, but its use has been employed infrequently for herpetofaunal control. The most publicized instances involved Brown Tree Snakes (*Boiga irregularis*) on Guam, where Brooks et al. (1998) tested lethality of dermal and oral drugs. Clark and Savarie (2012) and Clark et al. (2012) used and evaluated bait poisoning techniques. We are unaware of similar techniques being applied on invasive snakes in Middle America, but evaluating drug toxicity and establishing bait-poisoning plots for controlling *B. imperator* on Cozumel Island should be evaluated seriously for a long-term control or eradication program.

Invasive anuran control programs in Australia included spraying lethal chemicals in water sources (Kelehear et al. 2012). In the USA, Witmer et al. (2015) evaluated toxicity of different compounds on American Bullfrogs (*Lithobates catesbeianus*). Several species of anurans and other amphibians have evolved species-specific embryogenic suppression pheromones to reduce intraspecific competition, whereby older larvae produce substances that inhibit development of younger conspecifics (Tingley et al. 2017). The feasibility of using these embryogenic inhibitors for controlling invasive anurans in water sources has received recent attention, with some promising results (Clarke et al. 2016).

A key aspect for the success of some invasive anurans are potent chemical defenses that most vertebrate predators cannot tolerate. Nonetheless, several invertebrate groups are immune to those toxins, and many of them, such as dragonfly nymphs, fishing spiders, water beetles, ants, crabs, and crayfish, voraciously consume tadpoles or early stage metamorphs. Thus, introducing native invertebrate predators can be a biological control option when the only species in the water source is an alien anuran (Cabrera-Guzmán et al. 2012; Cabrera-Guzmán et al. 2015).

A major advantage for invasive species when reaching a new area is access to a parasite-free space (Torchin et al. 2003). With this notion in mind, it makes sense to introduce the relevant parasite into the area in order to diminish the fitness of the invasive population (Torchin et al. 2003; Finnerty et al. 2018). Even if the invasive species does not develop a parasitic disease, accumulation of sub-lethal effects can have significant consequences on overall performance, growth rates, and reproduction potential of infected toads, as reported by Finnerty et al. (2018). Another example of biological control used in Hawaii on the invasive *Eleutherodactylus coqui* was infecting them with the lungworm *Rhabdias elegans* (Marr et al. 2010).

A successful molecular tool for early detection and monitoring aquatic and semiaquatic invader species is testing water sources for their waterborne environmental DNA (eDNA), as described by Jerde et al. (2011) and Bohmann et al. (2014). So far, however, this technique has been used mainly for detecting rare or elusive species, although it was used to detect invasive amphibians (Dejean et al. 2012) and reptiles (Piaggio et al. 2014; Hunter et al. 2015). The advantage of eDNA is appealing because it can improve chances for detecting hard to observe species or to identify species displaying crypsis (Jerde et al. 2011; Piaggio et al. 2014). This method also allows conservation monitors to identify key amphibian breeding sites, and could be a valuable tool for locating strategic invasion places, such as those near airports, maritime ports, and plant nurseries (Tingley et al. 2017). In addition, it can be a complementary tool for post-eradication confirmation surveys.

A key feature for managing introduced species, especially in large areas, is to identify important sites to focus control and eradication efforts, as well as to prevent invasions and/or reinvasions before they occur. Environmental niche modelling (ENM) has been a helpful tool for identifying potential corridors among the sources and areas vulnerable to invasions (Peterson 2003; van Wilgen et al. 2009). In fact, the use of ENM for managing and controlling introduced herpetofaunal species recently has been increasing, with several examples originating in Middle America (Rödder et al. 2008; van Wilgen et al. 2009; Lira-Noriega and Ramírez 2016; Yáñez-Arenas et al. 2016).

Decision makers often require methods that help them justify and decide where, when, and on which species to target conservation and/or control programs. For these reasons, there exist protocols that can be used to determine if a species is potentially at risk and deserves attention. Generally, these protocols consist of a questionnaire that must be answered by a specialist or by a panel of experts; examples are revealed in “Método de Evaluación Rápida de Invasividad (MERI) para Especies Exóticas en México” (González-Martínez et al. 2017), and in the Harmonia+ and Pandora+ protocols for invasive species and invasive pathogens, respectively (D’hondt et al. 2015).

Finally, and most important of all, is the human component, which is pivotal for the success of any management program governing actions associated with invasive species (McNeely 2001; García-Llorente et al. 2008). The most obvious reason is that humans are often the determining factor when they transport species into new areas voluntarily or involuntarily, thereby enabling individual animals to cross biogeographical barriers (Vitousek et al. 1997; Mack et al. 2000). Personal attitudes of people relating to introduced species also strongly influence management preferences by giving more support to non-intervention approaches when they think animals have equal rights of existence versus control program when they consider human intervention acceptable for maintaining ecosystem integrity (Sharp et al. 2011). In addition, how urgent or harmful people perceive the risk of invasive species to be can directly reflect a potential willingness to help provide funding or other support for developing strategic management or control programs (García-Llorente et al. 2011). Therefore, any management policy should include serious sociological decisions, together with effective proclamation and educational campaigns incorporating biosecurity as a real value for local citizens.

## Conclusions

As noted previously, interest in introduced invasive species is taxonomically skewed toward other vertebrates, such as mammals, birds, or fishes. Thus, with a few exceptions, the ecological influences and damages caused by introduced invasive amphibians and reptiles are unknown. This lack of knowledge can have ominous consequences, such as taxonomic uncertainty, causing voids in legislation, and omissions of reptiles and amphibians in many biosecurity protocols or practices. Additionally, a great proportion of the literature on monitoring or control programs corresponds to technical reports not easily accessible to other researchers and/or managers. A first barrier to overcome is to encourage managers and researchers to identify results of monitoring and control programs on the invasive herpetofauna and to have that information published in accredited journals.

For conservationists to influence protocols, it is imperative that they promote stricter legislation on damaging practices, such as the pet trade, which should be discouraged, especially in those species with high potential of being harmful to Middle American ecosystems, even if they are already present, like *Trachemys* spp., or those that are common in the pet trade of a region, but not yet reported in the wild, such as *Varanus exanthematicus* and *Python molurus*. Stricter legislation should not be limited to vertebrate species, but also extended to pathogens associated with amphibians (or reptiles), such *Batrachochytrium dendrobatidis*, *Saprolegnia parasitica*, and *Ranavirus* spp. We are aware that such listing of potential harmful pathogens exists for Mexico (D.O.F. 2016), but it is unknown to us if any equivalent legislation exists in other Middle American countries.

Finally, the ever-growing trade of goods on a global scale, the increasing interest by people for keeping exotic pets, and the human persistence for environmental degradation will continue to favor arrival and settlement of invasive species. Regrettably, the frequency, scope, and intensity of biological invasions are expected to increase during the ensuing decades. Thus, the study and management of introduced amphibians and reptiles in Middle America is a topic that offers a wide spectrum of opportunities for career development associated with young researchers, conservationists, and other professionals dealing in ecological restoration.

We consider biological conservation as a human value, which includes a series of moral codes and behaviors that transcend time and culture and define us as a species. In this way, and like other values, it contributes to building prosperity and free coexistence among societies. It is also obvious that invasive species control and management is controversial, since many people put a high value on any single living organism, independent of its origin. If they accept invasive species to be a legitimate part of our ecological footprint, however, it is clear that we have a moral and ethical responsibility to educate them on the negative impacts invasive species have on the overall well-being of our biosphere. At the same time, it is also our responsibility to help maintain biosecurity and ecological restoration measures as advocates to prevent, mitigate, and remediate damages caused by invasive species until the majority of humanity accepts the fact that being good stewards of our living spaces is the right thing to do. Conservation professionals also must understand what motivates people’s different attitudes towards invasive species (e.g., why they transport them, whether they perceive them as harmful or not, or whether they are willing to accept control methods or not) in order to develop meaningful programs that discourage harmful behaviors and promote more responsible attitudes. Therefore, integration of the human and technical component is fundamental for accepting biosecurity as one more principle guiding societal behavior.

## Supplementary materials

Supplementary material 1 Multipoint shapefiles, occurrence records for introduced and translocated reptiles and amphibians in Mexico and Central American Countries Data type species data https://binary.pensoft.net/file/517840This dataset is made available under the Open Database License (http://opendatacommons.org/licenses/odbl/1.0/). The Open Database License (ODbL) is a license agreement intended to allow users to freely share, modify, and use this Dataset while maintaining this same freedom for others, provided that the original source and author(s) are credited.Víctor Hugo González-Sánchez, Jerry D. Johnson, Larry David Wilson
